# An Improved Football Team Training Algorithm for Global Optimization

**DOI:** 10.3390/biomimetics9070419

**Published:** 2024-07-08

**Authors:** Jun Hou, Yuemei Cui, Ming Rong, Bo Jin

**Affiliations:** 1 Faculty of Sports Science, Ningbo University, Ningbo 315211, China; 2011042026@nbu.edu.cn (J.H.); 2011042004@nbu.edu.cn (Y.C.); 2 Research Academy of Grand Health, Ningbo University, Ningbo 315211, China; 3 Institute of Systems and Robotics (ISR), Department of Electrical and Computer Engineering (DEEC), University of Coimbra, 3030-290 Coimbra, Portugal; jin.bo@isr.uc.pt

**Keywords:** football team training algorithm, metaheuristic algorithms, fitness distance-balanced strategy, non-monopoly extra training strategy, population restart strategy

## Abstract

The football team training algorithm (FTTA) is a new metaheuristic algorithm that was proposed in 2024. The FTTA has better performance but faces challenges such as poor convergence accuracy and ease of falling into local optimality due to limitations such as referring too much to the optimal individual for updating and insufficient perturbation of the optimal agent. To address these concerns, this paper presents an improved football team training algorithm called IFTTA. To enhance the exploration ability in the collective training phase, this paper proposes the fitness distance-balanced collective training strategy. This enables the players to train more rationally in the collective training phase and balances the exploration and exploitation capabilities of the algorithm. To further perturb the optimal agent in FTTA, a non-monopoly extra training strategy is designed to enhance the ability to get rid of the local optimum. In addition, a population restart strategy is then designed to boost the convergence accuracy and population diversity of the algorithm. In this paper, we validate the performance of IFTTA and FTTA as well as six comparison algorithms in CEC2017 test suites. The experimental results show that IFTTA has strong optimization performance. Moreover, several engineering-constrained optimization problems confirm the potential of IFTTA to solve real-world optimization problems.

## 1. Introduction

These optimization problems span the fields of scientific research, medical technology, and industrial applications, playing a crucial role in supporting the growing demand for artificial intelligence and machine learning [1,2,3,4,5]. The field of optimization focuses on finding the best solution among multiple alternatives to solve a problem. The goal is to maximize or minimize the objective function by adjusting the values of the decision variables, given the constraints. Model-based approaches to continuous and discrete global optimization [6] state that most real-world optimization problems are nonlinear, complex, indivisible, and involve many decision variables and constraints. For such optimization problems, it is usually best to use stochastic methods to deal with them. Metaheuristic algorithms are stochastic methods with good flexibility and strong adaptability and thus are widely used in the optimization field [7,8,9,10].

**Figure 1:**
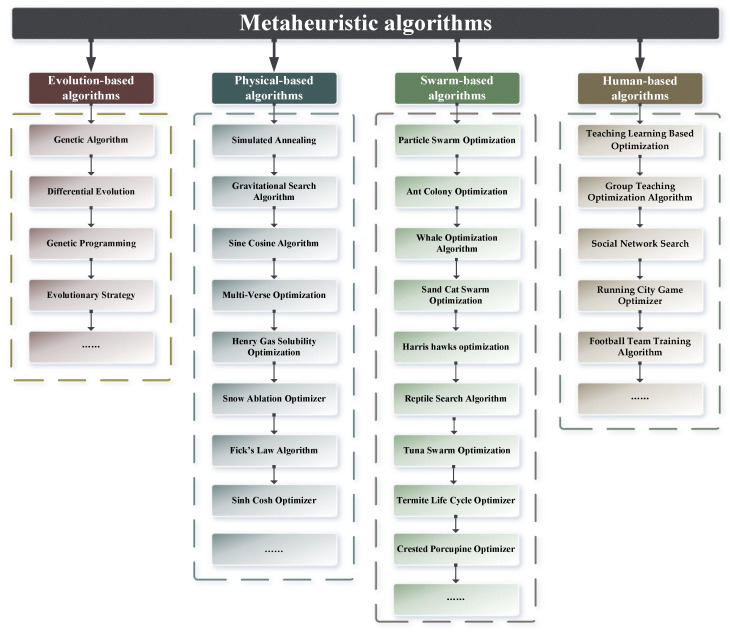
Classification of metaheuristic algorithms.

Metaheuristic algorithms are inspired by research in the fields of physicochemical laws of nature, biological behavioral mechanisms, and human social behavior. With the growth of metaheuristic algorithms, such algorithms are gradually divided into four categories [11]: evolution-based algorithms, physical-based algorithms, swarm-based algorithms, and human-based algorithms. Evolution-based algorithms contain the genetic algorithm (GA) [12], differential evolution (DE) [13], genetic programming (GP) [14], the evolutionary strategy (ES) [15], etc. Physical-based algorithms are derived from physicochemical laws or phenomena, such as simulated annealing (SA) [16], the gravitational search algorithm (GSA) [17], the sine cosine algorithm (SCA) [18], multi-verse optimization (MVO) [19], Henry gas solubility optimization (HGSO) [20], the snow ablation optimizer (SAO) [21], the Archimedes optimization algorithm (AOA) [22], Fick’s law algorithm (FLA) [23], the sinh cosh optimizer (SCO) [24], etc. Particle swarm optimization (PSO) [25] and ant colony optimization (ACO) [26] are the most famous swarm-based metaheuristic algorithms, and others include the whale optimization algorithm (WOA) [27], Harris hawks optimization (HHO) [28], sand cat swarm optimization (SCSO) [29], the reptile search algorithm (RSA) [30], tuna swarm optimization (TSO) [11], the termite life cycle optimizer (TLCO) [31], the crested porcupine optimizer (CPO) [32], and so on. Human-based metaheuristics construct algorithmic formulas by mimicking human behavioral habits, including teaching–learning-based optimization (TLBO) [33], the group teaching optimization algorithm (GTOA) [34], social network search (SNS) [35], the running city game optimizer (RCGO) [36], and so on. These algorithms mentioned above are given in Figure 1.

The algorithms mentioned above are all designed to find the optimal solution in the optimization domain, although they are of different types and are different in their sources of inspiration. The no-free-lunch theory [37] states that there is no one algorithm for all problems. Therefore, it is necessary to choose the most appropriate algorithm for a specific problem and to extend the applicability of the algorithm as much as possible.

The football team training algorithm (FTTA) is a novel metaheuristic algorithm inspired by the soccer team training method proposed by Tian et al. in 2024 [38]. The FTTA proposes a collective training phase, a group training phase, and an extra training phase to achieve a better optimization result. However, the FTTA will weaken the population diversity in the later stage, and it is easy to fall into the local optima and the convergence speed will become slower. Determining how to maintain the population diversity of the FTTA and improve its ability to get rid of local optimization is the research direction of this paper.

To address the above research problems, this paper presents an improved football team training algorithm (IFTTA). Aiming at the unreasonable training problem of the FTTA during the collective training phase, a fitness distance-balanced collective training strategy is proposed, which ensures a sufficient exploitation ability while having a certain global exploration ability simultaneously. A non-monopoly search strategy is introduced in the extra training stage, which improves the exploration performance of the algorithm at the initial stage by applying the perturbation of putting different degrees to the optimal individual while improving the exploitation ability of the algorithm at the later stage and preventing the algorithm from falling into the local optimum. In addition, a population restart strategy is designed to improve the convergence accuracy and population diversity of the algorithm.

In the experimental part, we make a comprehensive comparison between the IFTTA and the FTTA as well as six other algorithms. We evaluate the performance of the algorithms using 29 benchmark functions from CEC 2017. Statistical methods such as the Wilcoxon rank sum test and the Friedman test were used for the evaluation, and the convergence and stability of the FTTA were also analyzed. The performance of the FTTA and the validity of the improvement suggestions are thus verified.

The main contributions of this paper are as follows:

1. This paper presents an FTTA variant based on three strategies, named the IFTTA.
2. The introduction of the fitness distance-balanced strategy makes the collective training phase more reasonable and strikes a balance between the exploitation and exploration capabilities of the FTTA.
3. In the extra training phase, a non-monopoly search mechanism is integrated to improve the quality of the optimal agent, which effectively prevents the algorithm from falling into the local optimum and improves the overall global optimization performance of the algorithm.
4. The population restart mechanism improves the convergence accuracy and population diversity of the algorithm.
5. The performance of the IFTTA was compared with seven metaheuristic algorithms including the FTTA in different dimensions (Dim = 10, 30, 50, 100) using the CEC2017 benchmark function. The Wilcoxon rank sum test and the Friedman test were used for the evaluation, thus providing evidence of the efficiency of IFTTA and the validity of the suggested improvements. In addition, the optimal selection of each strategy of the IFTTA is investigated.
6. Several engineering-constrained optimization problems confirm the potential of the IFTTA to solve real-world optimization problems.

The paper is organized as follows: Section 2 describes the general overview of the FTTA. The details of the three improvement strategies included in the IFTTA are given in Section 3, along with a time complexity analysis. Section 4 and Section 5 are the experimental parts, which give the experimental results and analysis of the IFTTA in the CEC2017 test suite and engineering-constrained optimization problems. Finally, Section 6 summarizes the paper.

## 2. Football Team Training Algorithm (FTTA)

In this section, the mathematical model of the FTTA is presented, containing an initialization phase, a collective training phase, a group training phase, and an individual extra training phase.

### 2.1. Initialization Phase

In the FTTA, like other metaheuristic algorithms, population initialization is achieved by randomly generating N agents within the problem space. The formula is expressed as follows:

(1) $$X_{i}^{1}=lb+rand\times \left(ub-lb\right),i=1,2,\ldots ,N$$

where $X_{i}^{1}$ is the *i*th agent of the first generation. ub and lb denote the upper and lower boundaries of the problem space. N denotes the total number of agents used by the FTTA in the optimization process. rand represents a random number between 0 and 1. The initial population can be represented as follows:

(2) $$X=\left[\begin{array}{c} X_{1}\\ X_{2}\\ \vdots \\ X_{i}\\ \vdots \\ X_{N} \end{array}\right]=\left[\begin{array}{cccccc} X_{1,1} & X_{1,2} & \cdots & X_{1,j} & \cdots & X_{1,Dim}\\ X_{2,1} & X_{2,2} & \cdots & X_{2,j} & \cdots & X_{2,Dim}\\ \vdots & \vdots & \vdots & \vdots & \vdots & \vdots \\ X_{i,1} & X_{i,2} & \cdots & X_{i,j} & \cdots & X_{i,Dim}\\ \vdots & \vdots & \vdots & \vdots & \vdots & \vdots \\ X_{N,1} & X_{N,2} & \cdots & X_{N,j} & \cdots & X_{N,Dim} \end{array}\right]$$

where $X_{i,j}$ denotes the *j*th position of the *i*th solution, and Dim denotes the dimension size of the given problem.

### 2.2. Collective Training Phase

In the collective training phase, the FTTA randomizes agents into four categories: followers, discoverers, thinkers, and volatilities. In addition, all agents randomly select one of these types for the position update in each iteration.

For the agents that choose the follower role, they are randomly moved toward the optimal agent in each dimension with the following formula:

(3) $$X_{i}^{t+1}=X_{i}^{t}+rand\times \left(X_{best}-X_{i}^{t}\right)$$

where $X_{best}$ denotes the agent with the smallest fitness among all agents (in the case of the minimization problem). t denotes the current number of iterations.

For discoverers, these agents learn from both the best and the worst agents in the following way:

(4) $$X_{i}^{t+1}=X_{i}^{t}+rand\times \left(X_{best}-X_{i}^{t}\right)-rand\times \left(X_{worst}-X_{i}^{t}\right)$$

where $X_{worst}$ denotes the global worst agent.

Thinkers are different from discoverers in that they directly learn the gap between the optimal agent and the worst agent with the following formula:

(5) $$X_{i}^{t+1}=X_{i}^{t}+rand\times \left(X_{best}-X_{worst}\right)$$

Instead of learning from other agents, the volatiles train themselves in a way that is defined as follows:

(6) $$X_{i}^{t+1}=X_{i}^{t}\times \left(1+T\left(t\right)\right)$$

where $T\left(t\right)$ is a random number obeying a t-distribution with the number of iterations t as the degree of freedom.

### 2.3. Group Training Phase

During the group training phase, the FTTA will use mixed Gaussian expectation maximum (MGEM) adaptive clustering methods to categorize the agents into four identities: striker, midfielder, defender, and goalkeeper. In the FTTA, there are at least 2 agents of each category. When there are certain types of agents with less than 2, the FTTA will perform a uniform random grouping of all agents. After the grouping is completed, optimal learning, random learning, and random communication are executed randomly for all agents. The specific formula is expressed as follows:

(7) $$Optimal\;learning:X_{i}^{t}=\left\{\begin{array}{c} X_{i}^{t},if\;rand>p_{study}\\ X_{best},if\;rand\leq p_{study} \end{array}\right.$$

(8) $$Random\;learning:X_{i}^{t}=\left\{\begin{array}{c} X_{i}^{t},if\;rand>p_{study}\\ X_{random},if\;rand\leq p_{study} \end{array}\right.$$

(9) $$Random\;communication:X_{i}^{t}=\left\{\begin{array}{l} X_{i}^{t},if\;rand>p_{comm}\\ X_{random}\times \left(1+randn\right),if\;rand\leq p_{comm} \end{array}\right.$$

where $p_{study}$ denotes the learning probability. $p_{comm}$ denotes the communication probability. $X_{random}$ denotes a randomly selected individual. randn is a random number following a normal distribution.

### 2.4. Individual Extra Training Phase

After group training, the FTTA calculates the fitness of each agent. For each agent, the better fitness will be selected from the offspring and the parent. After that, the optimal agent is extra trained with the following formula:

(10) $$X_{i}^{t+1}=X_{i}^{t}\times \left(1+\left(1-1/t\right)\times Gauss+1/t\times Cauchy\right)$$

where Gauss and Cauchy are random numbers obeying Gaussian and Cauchy distributions, respectively. After the three phases of training, all agents first perform a boundary condition judgment, and the portion that exceeds the boundary will be reset to the boundary value. Then the fitness calculation is performed to obtain the best agent until the stopping condition is satisfied and the optimal agent and optimal fitness are output.

## 3. Proposed IFTTA

In this section, three improved strategies included in the IFTTA are described in detail: the fitness distance-balanced collective training strategy, the non-monopoly extra training strategy, and the population restart strategy. Furthermore, the time complexity of the proposed IFTTA and FTTA are analyzed to ensure that the proposed improvement algorithm in this paper enhances the performance of the basic algorithm without significantly increasing the time complexity.

### 3.1. Fitness Distance-Balanced Collective Training Strategy (FTS)

During the collective training phase in the FTTA, agents that choose the follower, discoverer, and thinker identities all learn from the best individual, which may lead to the premature convergence of the FTTA. In this paper, we consider that this is caused by the lack of exploration ability of the FTTA in the collective training phase. In order to overcome the shortcomings of the FTTA, this paper designs four collective training methods combining the roulette FDB (RFDB) method, the adaptive FDB (AFDB) method, the Cauchy FDB (CFDB) method, and the Gaussian FDB (GFDB) method, respectively. We will use the agent $X_{FTS}$ selected by the FTS method to replace $X_{best}$. The fitness distance-balanced (FDB) selection mechanism and its variants are presented below.

The FDB method mainly selects agents based on two parameters: the fitness of the agent and the distance between the agent and the best agent [39]. The FDB calculates the score of each agent in the population based on these two parameters. The higher the score of an agent, the better the quality of that agent. The FDB score is calculated as follows:

(11) $$Dis_{i}=\sqrt{{\left(X_{i,1}-X_{best,1}\right)}^{2}+{\left(X_{i,2}-X_{best,2}\right)}^{2}+\ldots +{\left(X_{i,Dim}-X_{best,Dim}\right)}^{2}}$$

(12) $$S_{i}=\omega \times norm\left(fitness_{i}\right)+\left(1-\omega \right)\times norm\left(Dis_{i}\right)$$

where Dim denotes the dimension of the agent. $norm\left(fitness_{i}\right)$ denotes the normalized value of fitness and $norm\left(Dis_{i}\right)$ denotes the normalized value of distance. ω is the weighting factor, which takes the value of 0.5 in FDB.

RFDB employs a roulette selection strategy that no longer singularly selects the agent with the first score, but instead determines the probability of selection for each agent based on the ratio of the individual agent’s score to the total score of all agents.

AFDB, CFDB, and GFDB all improve the selection of the FDB by adjusting the value taken by ω. AFDB presents an adaptive W updating method, shown below:

(13) $$\omega =0.4+\frac{\mathrm{mod}(t,t_{\max})}{t_{\max}}\times 0.6$$

where $\mathrm{mod}\left(\cdot \right)$ denotes the calculation rule for taking the remainder. CFDB and GFDB employ random numbers based on Cauchy and Gaussian distributions as weight ω, respectively. In summary, there are four FTTA algorithms combining four FDB variants that we named FTTA-RFDB, FTTA-AFDB, FTTA-CFDB, and FTTA-GFDB, respectively. These four variants will be evaluated in subsequent experiments to determine the optimal FDB variant.

### 3.2. Non-Monopoly Extra Training Strategy (NTS)

The quality of the optimal agent would affect the quality of the population updating, and if the optimal agent falls into a local optimum, this will lead to premature convergence of the algorithm. In order to avoid premature convergence and improve the global exploration ability, the optimal agent needs to be treated. The extra training phase of FTTA has perturbed the optimal agent, but it is less effective. The non-monopoly search strategy is a new local search method that modifies the dimension of the current solution space along the search space to further improve the quality of the optimal agent. In this paper, we propose a non-monopoly extra training strategy, which introduces the non-monopoly search strategy into the extra training stage and combines Gaussian and Cauchy operators to further improve the performance. The specific formula is as follows:

(14) $$X_{best,j}^{new}=rand\times X_{best,random}$$

(15) $$X_{best,j}^{new}=X_{best,j}-\left(X_{best,random}\times rand\right)\times eps-\left(X_{best,j}-1\right)$$

where $X_{best,j}$ denotes the *j*th dimension of the optimal agent and $X_{best,random}$ denotes the random dimension of optimal agent, ranging from 1 to Dim. Equation (14) is executed in the early stage and Equation (15) is employed in the post period. In this paper, Gaussian and Cauchy operators from the original extra training phase are introduced into the non-monopoly search strategy. The Cauchy operator effectively provides the agent with a wide range of perturbations, while the Gaussian operator provides the agent with finer tuning. As described in Equations (16) and (17) and Table 1, this paper provides details of nine FTTA variants that incorporate different non-monopolized extra training strategies.

(16) $$X_{best,j}^{new}=Select\left\{rand,Cauchy,Gaussian\right\}\times X_{best,random}$$

(17) $$X_{best,j}^{new}=X_{best,j}-\left(X_{best,random}\times rand\right)\times eps-\left(X_{best,j}-1\right)\times Select\left\{Cauchy,Gaussian\right\}$$

where $Select\left\{rand,Cauchy,Gaussian\right\}$ denotes the operator from the three operators. $Select\left\{Cauchy,Gaussian\right\}$ denotes the operator from the two operators.

### 3.3. Population Restart Strategy (PRS)

The agents of the FTTA are prone to falling into local optimality after multiple trainings. Therefore, this paper proposes a population restart strategy to enrich the population diversity. In PRS, we record the number of times $Trial_{i}$ that each agent does not get better after updating. If the adaptation of that agent does not improve after each update, Trial is added by 1. When the Trial value of an agent is greater than a certain threshold, PRS will be performed on that agent. The updating formula of PRS is expressed as follows:

(18) $$X_{i}^{t+1}=\left\{\begin{array}{l} X_{i}^{t}+{(1-\frac{t}{t_{\max}})}^{\frac{2t}{t_{\max}}}\times \left(lb+rand\times \left(ub-lb\right)\right)\times U,rand\leq 0.2\\ X_{i}^{t}+\left(0.2\times (1-rand)+rand\right)\times (X_{r1}^{t}-X_{r2}^{t}),rand>0.2 \end{array}\right.$$

where U is a binary vector including 0 or 1. When a random vector from 0 to 1 is generated and is less than 0.2, the array is changed to 0, and vice versa. $X_{r1}^{t}$ and $X_{r2}^{t}$ are two randomly selected agents in FTTA. After the execution of PRS, when the fitness of this agent improves, the value of $Trial_{i}$ is changed to 0 and the counting is restarted.

### 3.4. Implementation Steps of the IFTTA and Computational Time Complexity

In summary, the pseudo-code of the proposed IFTTA is shown in Algorithm 1.
**Algorithm 1** Improved football team training algorithm (IFTTA)1: Initialization: *t* = 0, *t_max_*, *N*. Generate an initial population randomly
2: while (*t* < *t_max_*) do
3:   Calculate the fitness values of each *X*_i_ to obtain *X*_best_ and *X*_worst_
4:   For *i* = 1:*N*
4:     Select *X*_FTS_ using FTS method according to Equation (12)
5:     Collective training: update each football player X_i_ according to Equations (3)–(6)
6:     Group training: update each football player X_i_ according to Equations (7)–(9)
7:     Calculate the fitness values of each *X*_i_ and *Trial*_i_
8:   End for
9:   Find the best player *X*_best_
10:  Individual extra training: update the best player *X*_best_ using NTS method according to Equations (16) and (17)
11:  For *i* = 1:*N*
12:    Update each football player *X*_i_ using PRS according to Equation (18)
13:  End for
14:  Calculate the fitness values of each *X*_i_ to obtain *X*_best_
15:  *t* = *t* + 1
14: end while


In the IFTTA, if the number of populations is *N*, the dimension is *D* and the maximum number of iterations is *T*. The time complexity of the population initialization phase is $O\left(N\times D\right)$. The time complexity of collective training with FTS and group training is $O\left(N\times D\times T\right)$. The time complexity of individual extra training with NTS is $O\left(D\times T\right)$. The time complexity of the PRS method is $O\left(\max\left(N\right)\times D\times T\right)$. In conclusion, the time complexity of the IFTTA is $O\left(T\times D\times \left(N+1+\max\left(N\right)\right)\right)$.

## 4. Numerical Experiments Based on CEC 2017 Test Suite

### 4.1. Experimental Environment and Parameter Setting

The experimental studies were implemented in MATLAB^®^ R2021b and run on AMD R9 7900X @ 4.70 GHz, 32 GB RAM, and x64-based processor. Three basic algorithms and three improved algorithms are selected for comparison in this paper, and the parameter settings of the competitors are shown in Table 2.

### 4.2. Test Functions and Performance Metrics

To comprehensively examine the performance of IFTTA, by comparing it with the competitors mentioned in Table 2, single-objective optimization tests are conducted in this paper using the CEC 2017 test suite in different dimensions (Dim = 10/30/50/100). In this paper, the experimental parameters are set to a population size of 50 and a maximum number of iterations of 1000. Each algorithm is run independently 51 times for each problem to improve the reliability of the statistical analysis. The details of the CEC2017 test suite are shown in Table 3 and can be found in the literature [46].

In the paper, we will record the best value, mean value, and standard deviation obtained by each algorithm when solving each test function. We use the Wilcoxon rank sum test and the Friedman test to analyze the performance of the IFTTA and the comparison algorithms on the CEC2017 test suite. Specifically, the Wilcoxon rank sum test is used to test whether there is a significant difference between the IFTTA and its competitors in terms of the performance of each function, which in turn determines whether the IFTTA is superior or inferior to its competitors. In addition, the final rankings of the algorithms on all functions from the Friedman test can also be used to assess the significant differences in overall performance between the algorithms. A 5% significance level was used in the statistical tests.

### 4.3. Determine the Best FTS Method on Test Suite

This subsection will determine the best FTS method. The FTTA-RFDB, FTTA-AFDB, FTTA-CFDB, FTTA-GFDB, and the basic FTTA proposed in Section 3.1 were experimented in the CEC2017 test suite and statistically analyzed using the Wilcoxon rank-sum test and the Friedman test. The Friedman test results for these algorithms are given in Table 4. The scores of the best-performing algorithms in each dimension are marked in bold in Table 4. In addition, the last row shows the average ranking of each algorithm for each of the four dimensions, and the last column gives the *p*-value of the Friedman test for each dimension.

**Figure 2:**
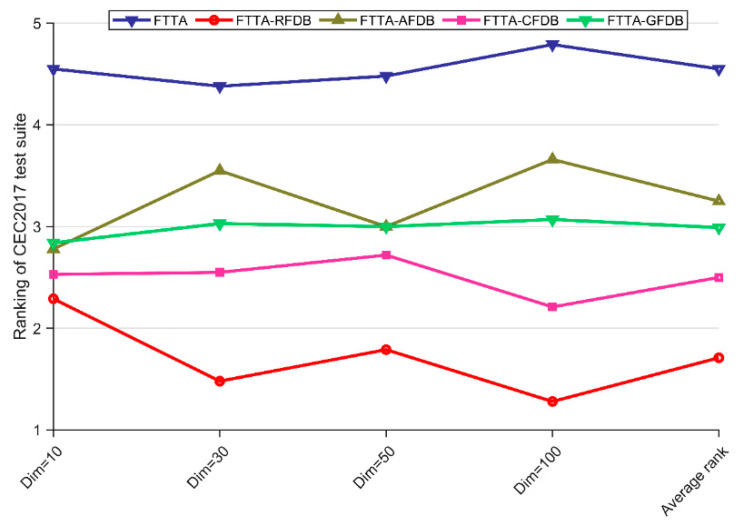
The rankings of FTTA and FTTA-FTS variants according to the Friedman test.

As can be seen from Table 4, all four variants of the FTTA proposed in this paper combining FTS methods show better performance than the basic FTTA. The FTTA-RFDB achieved the best scores in all the experiments, which suggests that the RFDB method is the best FTS method. The *p*-values of the Friedman test are less than 0.05, which indicates that all the variants are different from the FTTA. In conclusion, the results of the Friedman test reveal that the four variants combining FTS methods show better convergence performance compared to the basic FTTA. The rankings of FTTA and FTTA-FTS variants are visually shown in Figure 2.

The results of the Wilcoxon rank sum test for the two-by-two pairwise comparisons of the FTTA and the FTTA variants of the four integrated FTS methods are given in Table 5. The symbols “+”, “−”, and “=” indicate that the FTTA variants are superior, inferior, or similar to the basic FTTA. As can be seen in Table 5, the basic FTTA achieves a victory over its competitors on up to three functions in a single experiment. The FTTA-RFDB performs better with increasing dimensionality and outperforms the FTTA more often. The other three FTTA variants also beat the basic FTTA in more than half of the functions. In summary, the two statistical analyses clearly show that the FTS methods help the FTTA to strike a balance between exploration and exploitation and boost the performance of the FTTA, with the FTTA-RFDB showing the biggest boost. Thus, this paper will follow up with the use of RFDB as an FTS method for integration into the FTTA.

### 4.4. Determine the Best NTS Method on Test Suite

In this subsection, we evaluate the FTTA-NTS variants given in Table 1 and select the best NTS method. The Friedman test scores and the Wilcoxon rank sum test results for two-by-two comparisons for each algorithm are given in Table 6 and Table 7, respectively.

**Figure 3:**
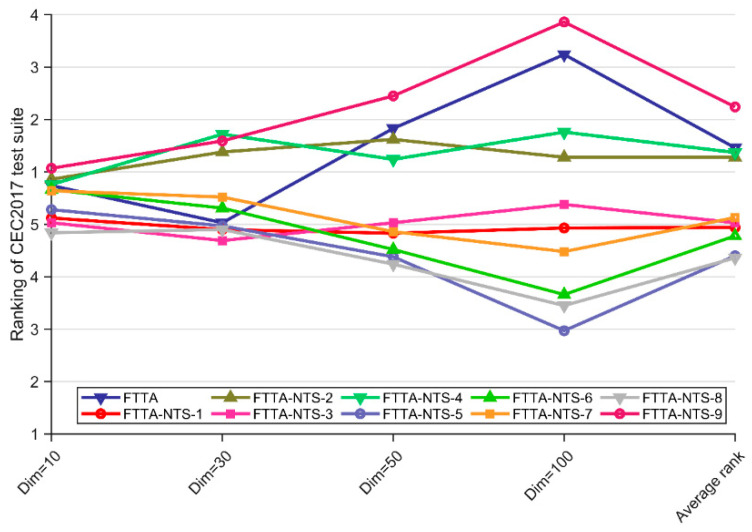
The rankings of FTTA and FTTA-NTS variants according to the Friedman test.

As can be seen from Table 6, the Friedman *p*-values for all dimensions except Dim = 10 are less than 0.05, which indicates that there is a significant difference in performance between the FTTA-NTS variant and the basic FTTA. Specifically, FTTA-NTS-3 obtains the best score on Dim = 30. FTTA-NTS-5 ranks first on Dim = 100. FTTA-NTS-8 scores the highest on Dim = 10 and Dim = 50. This indicates that the NTS strategy is effective in enhancing FTTA performance. The rankings of FTTA and FTTA-NTS variants are visually shown in Figure 3. It is noteworthy that the NTS-9 method without combining Gaussian and Cauchy operators failed to perform as well as the basic FTTA, and the rest of the NTS methods combining Gaussian and Cauchy operators outperformed the basic FTTA, which suggests that there is a need to improve the basic non-monopoly search method.

Based on Table 7, it can be concluded that the NTS method has limited enhancement for FTTA at Dim = 10 and Dim = 30. The NTS method can significantly improve the FTTA performance at Dim = 50 and Dim = 100. This is because NTS adopts the idea of dimension-by-dimension updating, which is more conducive to retaining the superior dimensions in higher dimensions. In summary, the FTTA variant using the NTS method performs better than the FTTA in most cases, with fewer cases of inferiority, and the NTS method is an effective method.

### 4.5. Strategies Effectiveness Analysis

**Figure 4:**
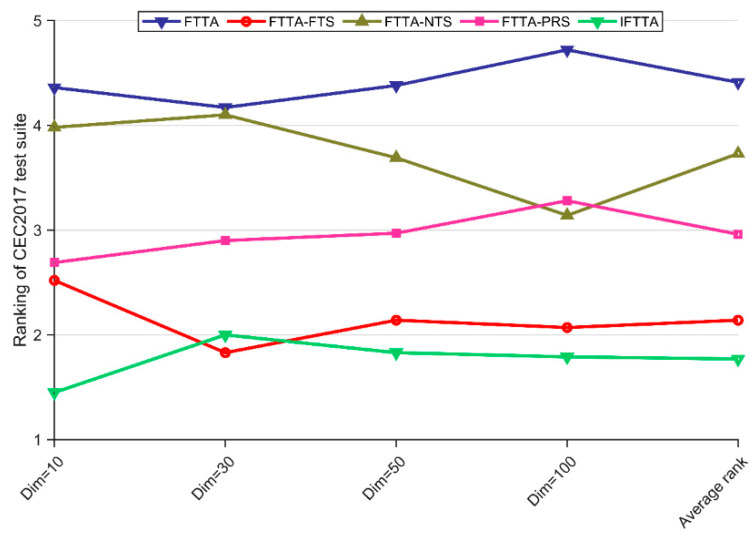
The rankings of FTTA and IFTTA variants according to the Friedman test.

It is necessary to evaluate the impact of the three improvement strategies proposed in this paper on FTTA. In this subsection, strategy effectiveness analysis will be performed. FTTA and FTTA-FTS, FTTA-NTS, and FTTA-PRS, as well as the IFTTA integrating the three strategies, are examined in experimental research. The Friedman test and the Wilcoxon rank sum test are employed to analyze the test results. The Friedman scores for the IFTTA and its three variants are given in Table 8 with a significance level of a = 0.05. The *p*-value of the Friedman test for all four dimensions is less than 0.05, which indicates that there is a significant difference between IFTTA with the three IFTTA variants as well as FTTA. Figure 4 visualizes the scores of IFTTA and the three variants. As can be seen in Figure 4, IFTTA performs best in the metric of average ranking, which indicates that although IFTTA is worse than FTTA-FTS in Dim = 30, it beats all variants in terms of overall performance. It is noteworthy that all variants have higher scores than the basic FTTA, suggesting that all improvement strategies are effective in enhancing FTTA.

Table 9 summarizes the results of the Wilcoxon rank sum test. The Total column in Table 9 shows that there are more “+” than “−” for IFTTA and all variants over FTTA, which implies that all strategies improve FTTA performance, and that IFTTA integrating all three strategies has the best overall performance.

### 4.6. Comparison with Other Algorithms

**Figure 5:**
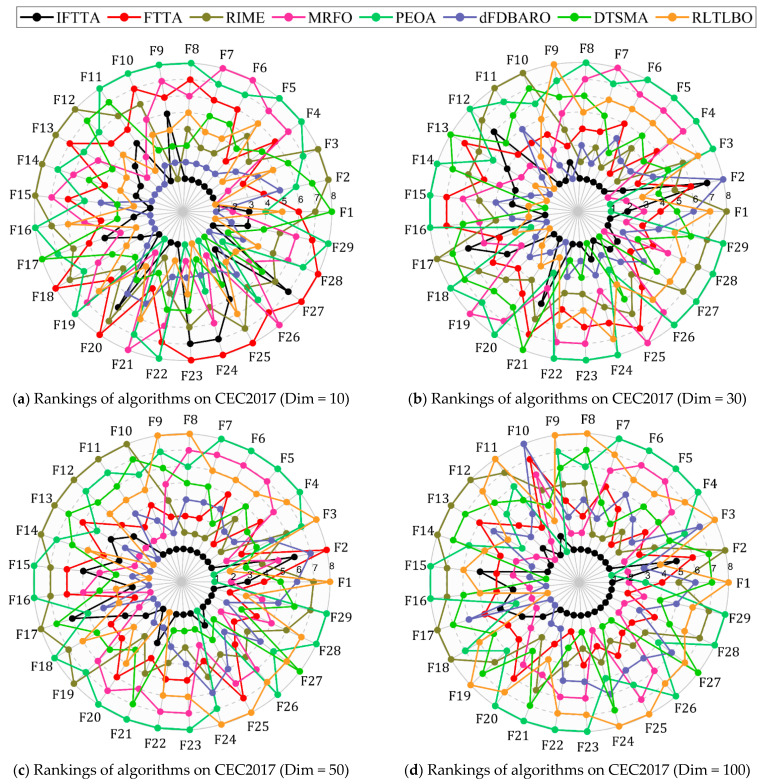
Rankings based on “Mean” of IFTTA and six comparison algorithms on each function.

In this subsection, the IFTTA is evaluated using the CEC 2017 test suite and is compared with six algorithms. The six algorithms include a physics-based algorithm RIME, a swarm-based algorithm MRFO, a human-based algorithm PEOA, and three improved algorithms. The detailed results obtained for IFTTA, FTTA, RIME, MRFO, PEOA, dFDBARO, DTSMA, and RLTLBO are shown in Table A1, Table A2, Table A3 and Table A4 in Appendix A. In order to show the performance for each function based only on the mean values, the spider plots based on the ranking of the mean values from Table A1, Table A2, Table A3 and Table A4 are exhibited in Figure 5. From Figure 5, it can be roughly concluded that IFTTA outperforms the competitors in all dimensions except the 10 dimension and performs similarly to dFDBARO in the 10 dimension. In the next analysis, the experimental results will be analyzed using the Friedman test and Wilcoxon rank sum test to avoid inaccurate conclusions caused by relying only on the mean analysis.

#### 4.6.1. Analysis Using the Wilcoxon Rank Sum Test

In this section, the results obtained by the IFTTA and the competitor in 51 runs were analyzed using the Wilcoxon rank sum test with a confidence level of 0.05, which are recorded in Table 10, where “+” indicates that the IFTTA outperforms the competitor, “−” denotes that the IFTTA is inferior to the comparison algorithm, and “=” denotes that IFTTA’s performance is statistically similar to the competitors.

By comparing the number of ‘+’ and ‘−’ in different cases, the total of ‘+’ is higher than ‘−’. This shows that IFTTA outperforms its competitors, and the details of the analysis are as follows:

(a) On Dim = 10, IFTTA outperforms (underperforms) FTTA on 27 (0) benchmark functions, RIME on 24 (3) benchmark functions, MRFO on 18 (3) benchmark functions, PEOA on 23 (2) benchmark functions, dFDBARO on 12 (9) benchmark functions, DTSMA on 20 (4) benchmark functions, and RLTLBO on 18 (3) benchmark functions. It can be concluded that the IFTTA outperforms six comparison algorithms on Dim = 10.
(b) On Dim = 30, IFTTA outperforms (underperforms) FTTA on 22 (1) benchmark functions, RIME on 25 (1) benchmark functions, MRFO on 17 (3) benchmark functions, PEOA on 23 (4) benchmark functions, dFDBARO on 20 (4) benchmark functions, DTSMA on 23 (2) benchmark functions, and RLTLBO on 18 (3) benchmark functions. It can be concluded that the IFTTA outperforms six comparison algorithms on Dim = 30.
(c) On Dim = 50, IFTTA outperforms (underperforms) FTTA on 22 (1) benchmark functions, RIME on 26 (1) benchmark functions, MRFO on 18 (3) benchmark functions, PEOA on 25 (3) benchmark functions, dFDBARO on 18 (1) benchmark functions, DTSMA on 25 (1) benchmark functions, and RLTLBO on 20 (4) benchmark functions. It can be concluded that the IFTTA outperforms six comparison algorithms on Dim = 50.
(d) On Dim = 100, IFTTA outperforms (underperforms) FTTA on 26 (0) benchmark functions, RIME on 28 (0) benchmark functions, MRFO on 20 (1) benchmark functions, PEOA on 25 (2) benchmark functions, dFDBARO on 22 (3) benchmark functions, DTSMA on 27 (1) benchmark functions, and RLTLBO on 26 (2) benchmark functions. It can be concluded that the IFTTA outperforms six comparison algorithms on Dim = 100.

#### 4.6.2. Analysis Using the Friedman Test

The Friedman test was used to further illustrate the performance differences between IFTTA and the competitors. The Friedman test results for the seven algorithms with a significance level of 0.05 are presented in Table 11.

**Figure 6:**
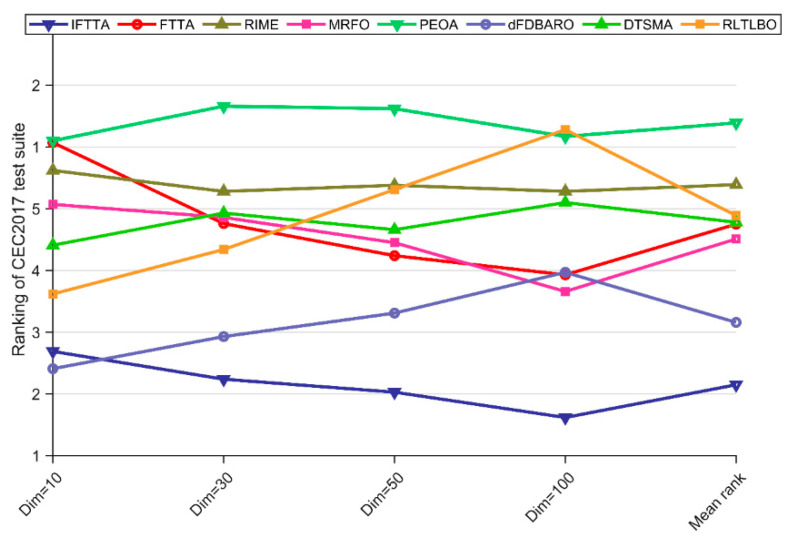
The rankings of FTTA and competitors according to the Friedman test.

As can be seen in Table 11, the Friedman *p*-values for all dimensions except Dim = 10 are less than 0.05, which indicates that there is a significant difference between the performance of the IFTTA and the other competitors on the CEC2017 test suite except Dim = 10. Figure 6 visualizes the Friedman scores obtained by the seven algorithms.

For Dim = 10, IFTTA ranks second behind dFDBARO, followed by RLTLBO, DTSMA, MRFO, and RIME, with PEOA and FTTA both occupying last place. For Dim = 30, IFTTA is ranked first, with dFDBARO and RLTLBO in second and third place, followed by FTTA, MRFO, DTSMA, RIME, and PEOA. For Dim = 50, IFTTA, FTTA, and dFDBARO are in the top three, followed by MRFO, DTSMA, RLTLBO, and RIME, and for Dim = 100, IFTTA is ranked first, followed by MRFO, FTTA, dFDBARO, DTSMA, RIME, and PEOA. In terms of “average ranking”, the IFTTA is ranked first with a Friedman score of 2.15. In summary, the Friedman test concluded that the IFTTA outperformed the other competitors in the CEC2017 test suite.

**Figure 7:**
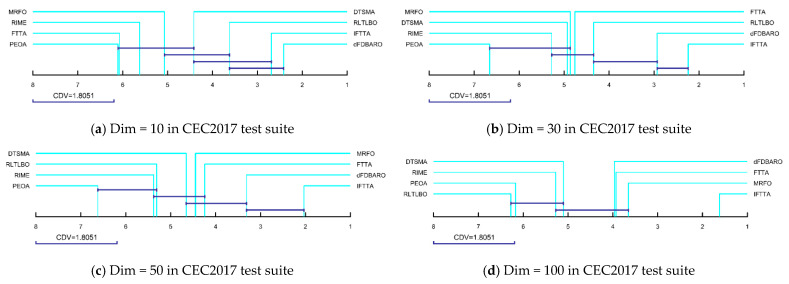
Multiple comparisons using the post-hoc Iman–Davenport test.

Moreover, the post-hoc Iman–Davenport test was used to further analyze the magnitude of the differences. The Iman–Davenport test is a statistical test based on the F-distribution with *K* − 1 and (*K* − 1)(*Num* − 1) degrees of freedom where *K* is the number of algorithms and N is the total number of functions in the test suite. For CEC2017, *K* = 8 and *Num* = 29. The Nemenyi test is used for post-hoc testing and the critical difference value (*CDV*) is used to determine the difference between the eight algorithms according to the Friedman scores. The *CDV* is calculated as expressed below:

(19) $$F_{F}^{2}=\frac{\left(Num-1\right)\times \chi_{F}^{2}}{Num\times \left(K-1\right)-\chi_{F}^{2}}$$

(20) $$CDV=q_{a}\times \sqrt{\frac{K\times \left(K+1\right)}{6\times Num}}$$

where $q_{a}$ is obtained from the F-distribution. For CEC2017, $q_{a}$ is 3.03 and CDV is 1.81. Figure 7 shows multiple comparisons of the magnitude of differences between IFTTA and the seven competitors, where there is no significant difference between the algorithms linked together by *CDV*. As shown in Figure 7, IFTTA significantly outperforms DTSMA, MRFO, RIME, FTTA, and PEOA at Dim = 10 and is not significantly different from RLTLBO and dFDBARO. At Dim = 30/50, there is no significant difference between the IFTTA and dFDBARO, and the IFTTA is significantly superior to the other competitors. At Dim = 100, the IFTTA obviously beats all the compared algorithms. In conclusion, according to the Wilcoxon rank sum test and Friedman test, the IFTTA proposed in this paper is superior to six comparison algorithms on the CEC2017 test suite.

#### 4.6.3. Analysis of Convergence Rate

**Figure 8:**
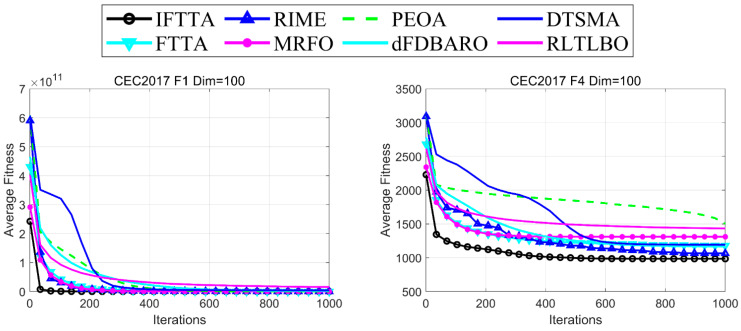

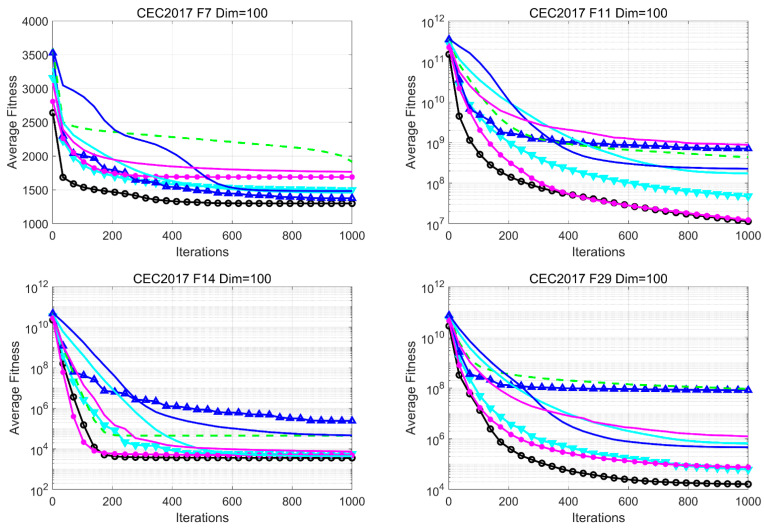
Convergence curves of IFTTA and competitors on six functions (Dim = 100).

**Figure A1:**
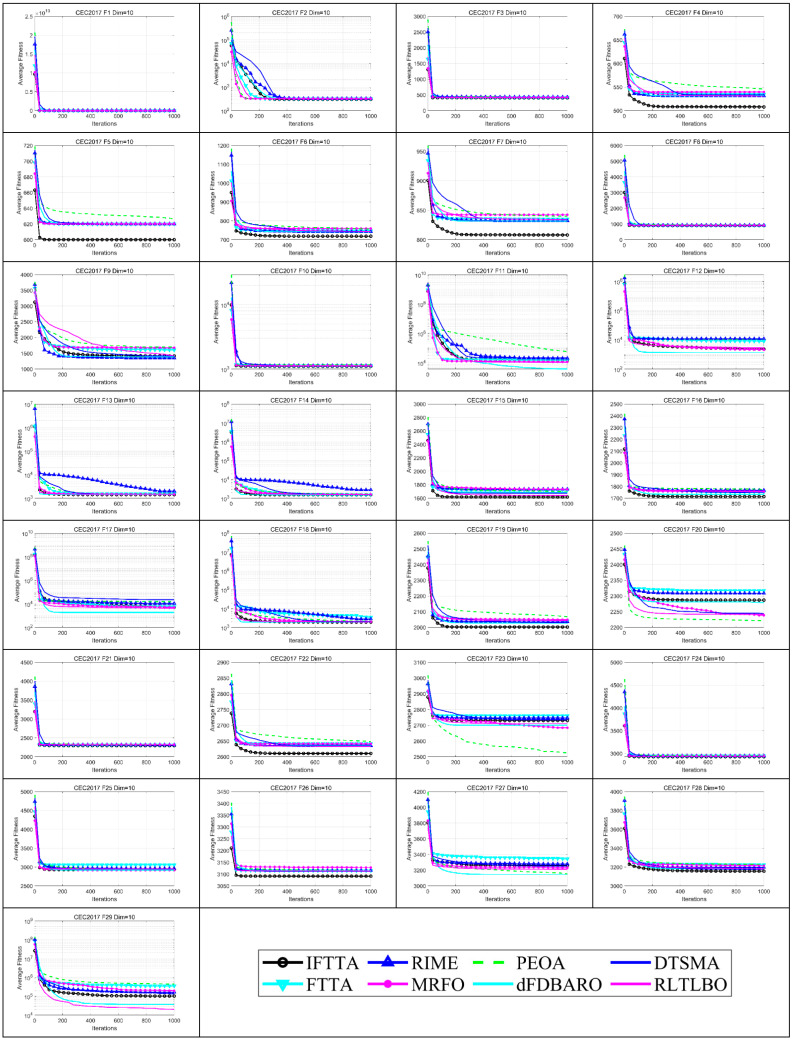
The convergence curves of IFTTA and competitors for CEC 2017 (Dim = 10).

**Figure A2:**
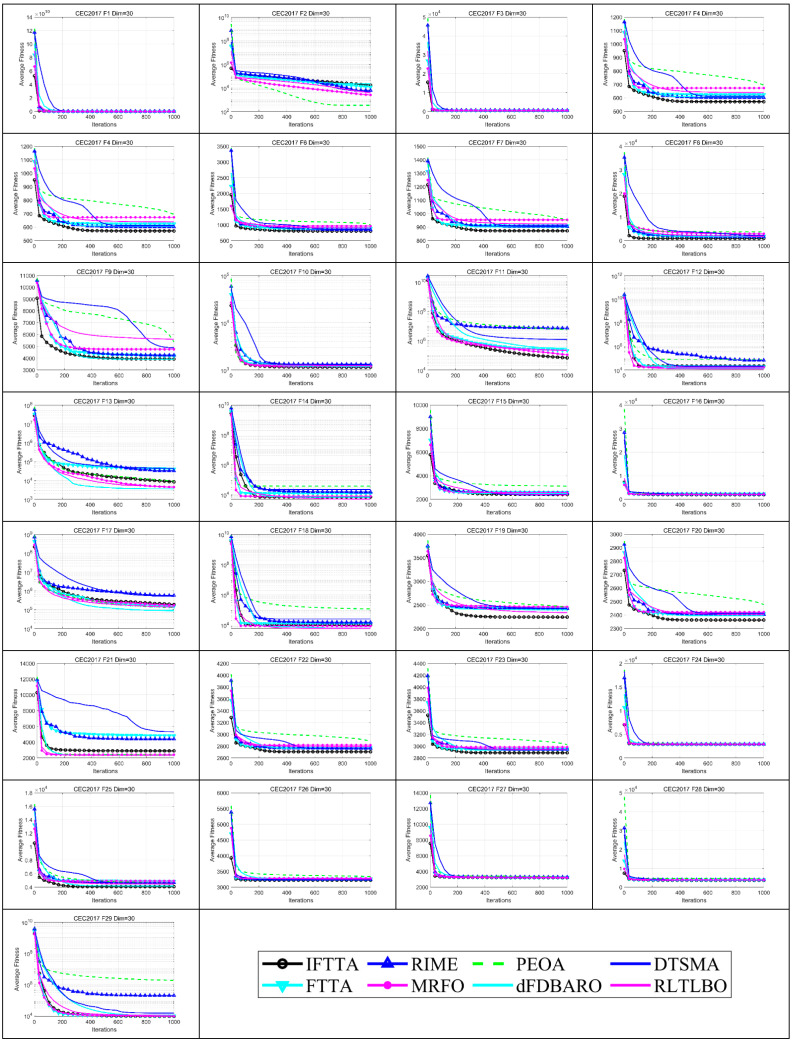
The convergence curves of IFTTA and competitors for CEC 2017 (Dim = 30).

**Figure A3:**
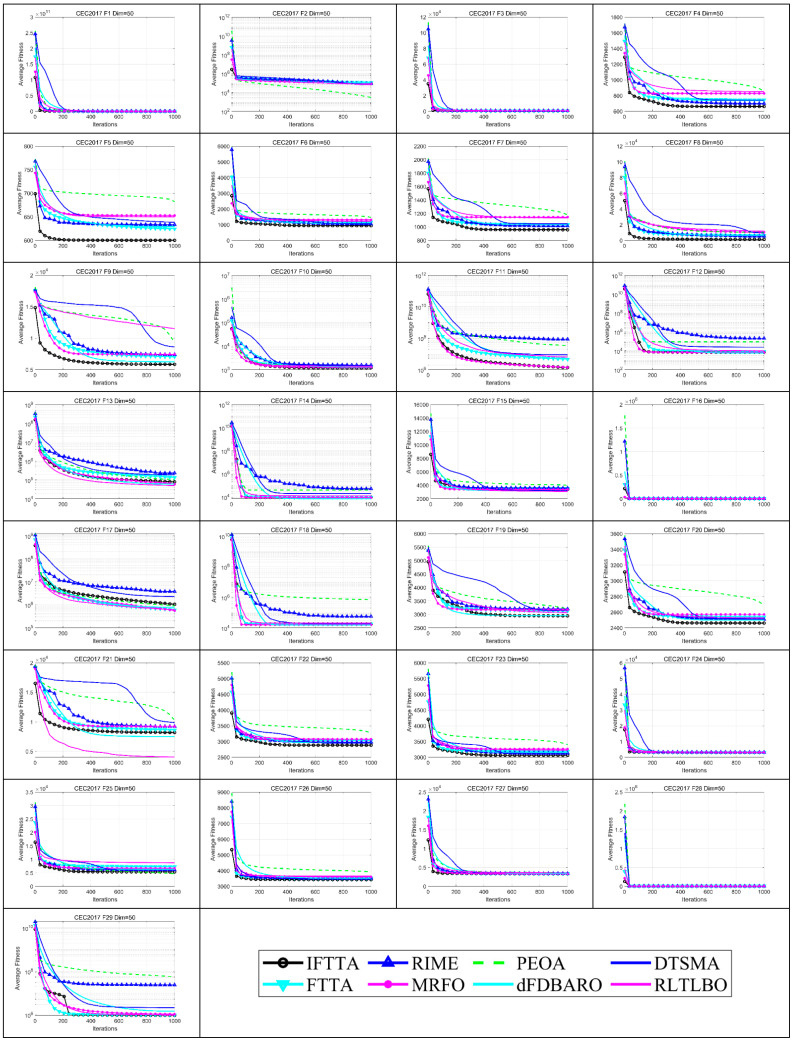
The convergence curves of IFTTA and competitors for CEC 2017 (Dim = 50).

**Figure A4:**
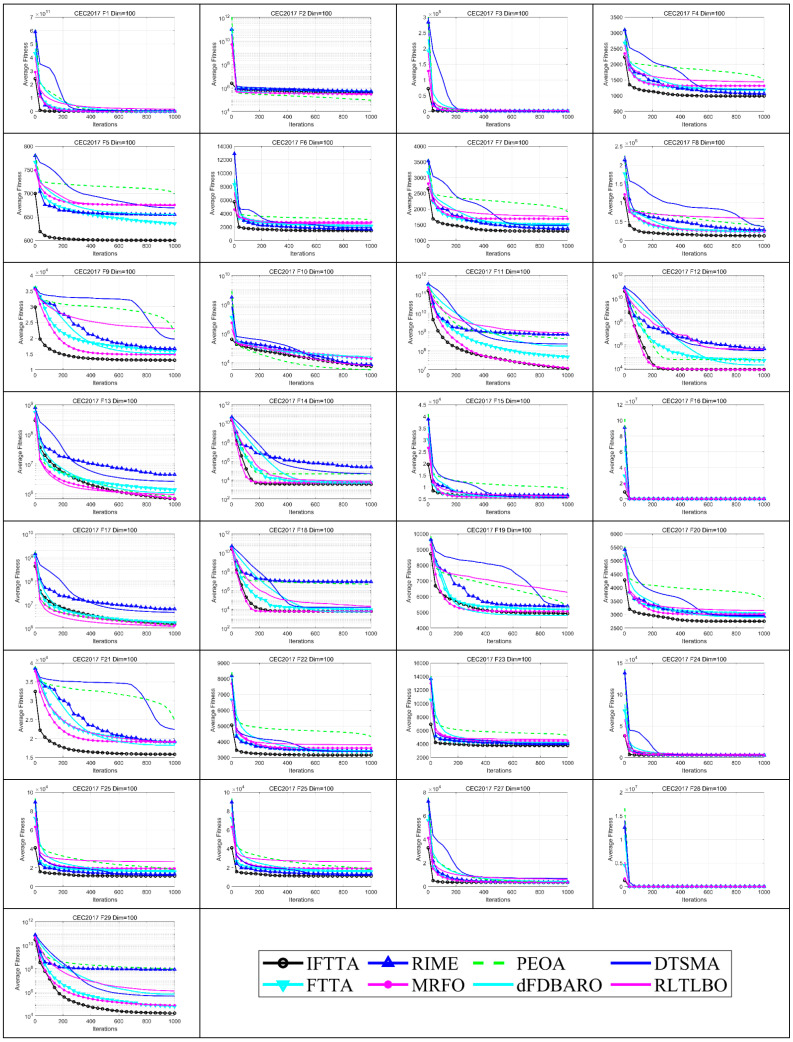
The convergence curves of IFTTA and competitors for CEC 2017 (Dim = 100).

In this section, the average convergence plots of 51 independent results are used to evaluate the convergence performance of the IFTTA and the competitors. For simplicity, the 100-dimensional unimodal function F1, the multimodal functions F4 and F7, the hybrid functions F11 and F14, and the composite function F29 are selected for presentation in Figure 8. The remaining convergence images are available in Figure A1, Figure A2, Figure A3 and Figure A4 in Appendix A. Figure 6 shows that the convergence performance of the IFTTA on all six functions is better than the competitors with faster convergence speed and convergence accuracy.

#### 4.6.4. Analysis of Robustness

**Figure 9:**
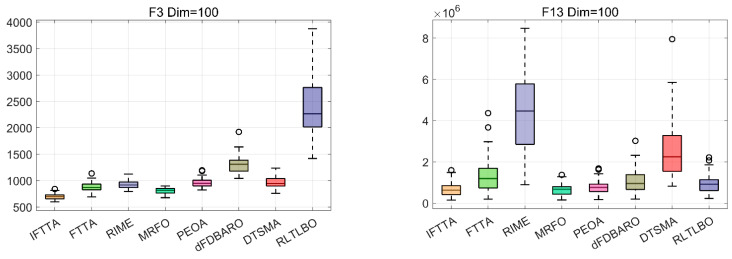

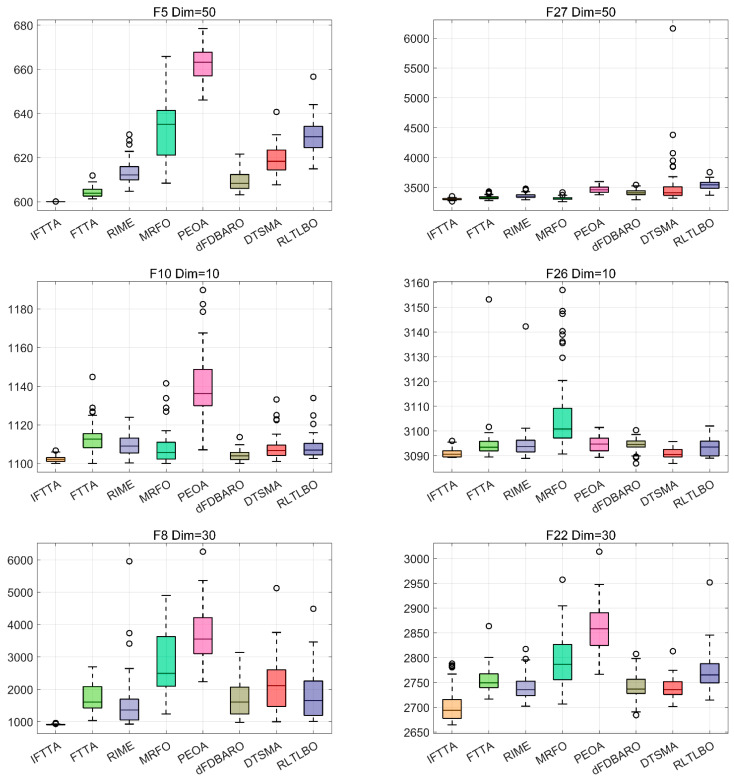
Box plots of IFTTA and competitors on eight functions.

**Figure A5:**
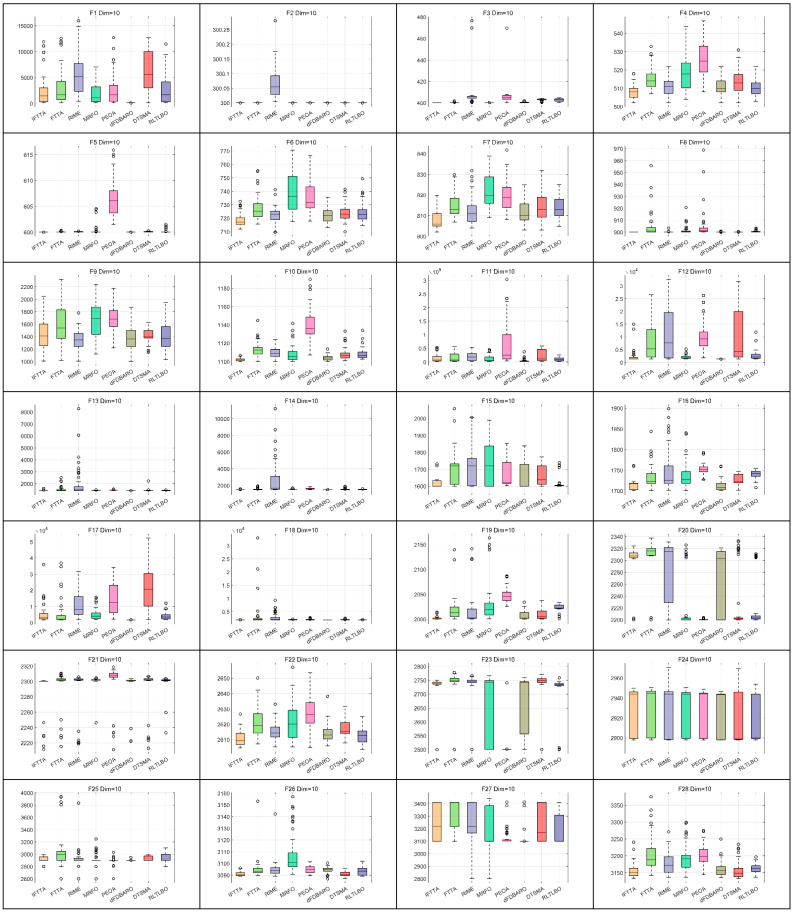
The box plots of IFTTA and competitors for CEC 2017 (Dim = 10).

**Figure A6:**
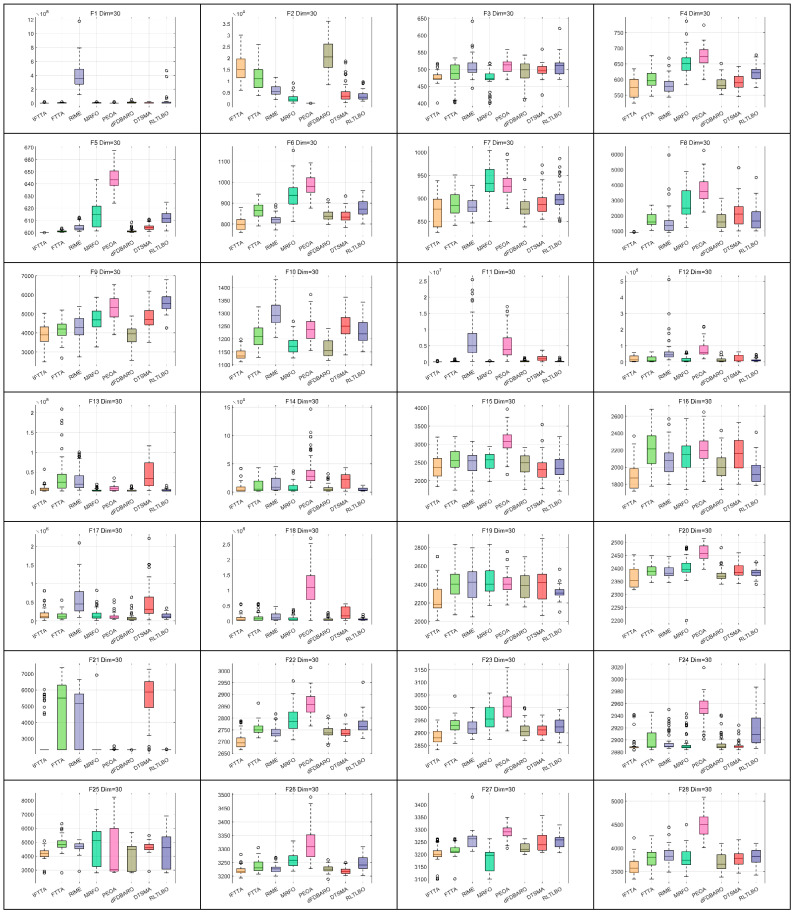
The box plots of IFTTA and competitors for CEC 2017 (Dim = 30).

**Figure A7:**
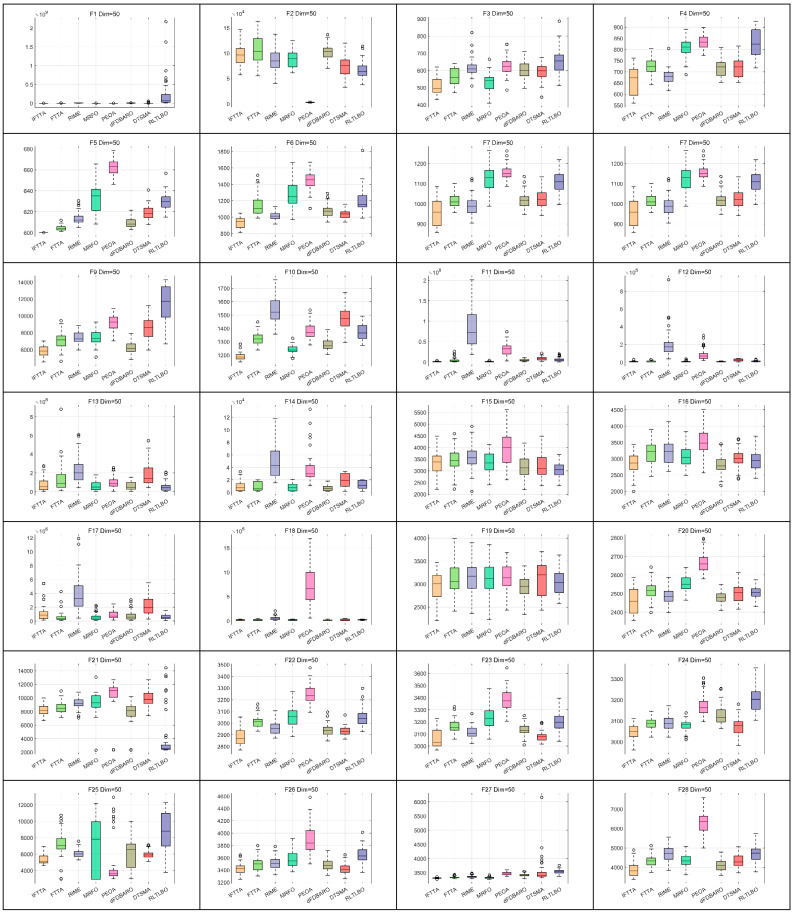
The box plots of IFTTA and competitors for CEC 2017 (Dim = 50).

**Figure A8:**
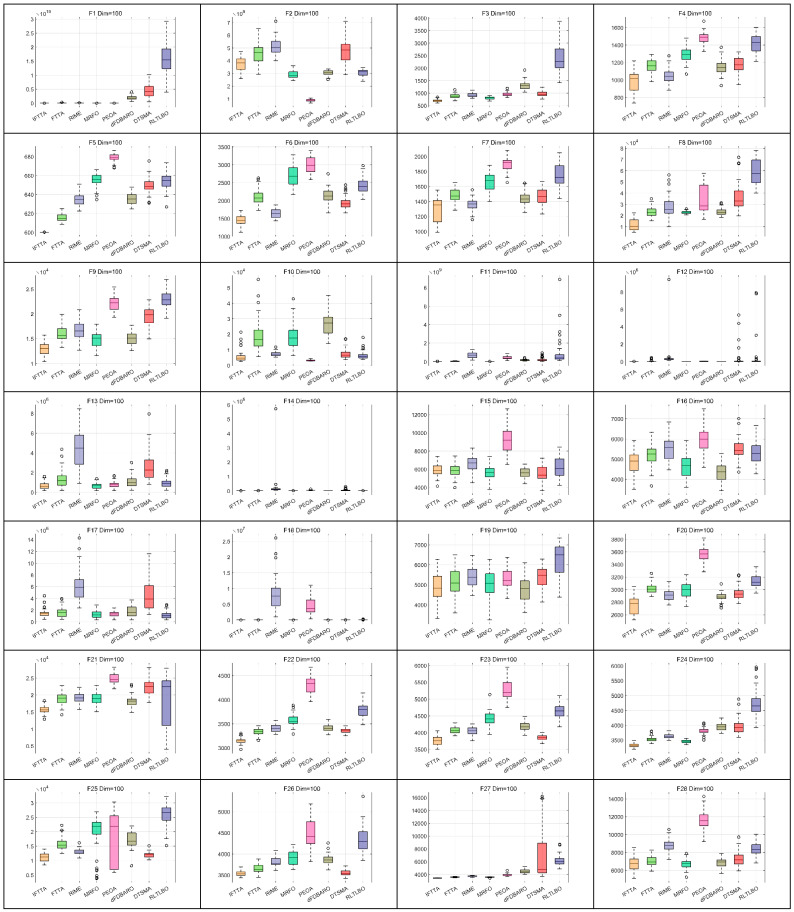
The box plots of IFTTA and competitors for CEC 2017 (Dim = 100).

In this subsection, box plots are used to evaluate the robustness of IFTTA. In box diagrams, narrower boxes indicate more concentrated results, and lower box positions indicate better results. Circles indicate bad values, i.e., solutions that are far from the centralized region. Figure 9 illustrates the distribution of the results from the IFTTA and the competitors on some functions. The rest of the images can be obtained in Figure A5, Figure A6, Figure A7 and Figure A8 in Appendix A. Figure 9 shows two functions from each dimension separately, and we can see that the boxes of the IFTTA are denser and have lower positions, which indicates that the IFTTA has better robustness.

## 5. Engineering-Constrained Optimization Problems

**Figure 10:**
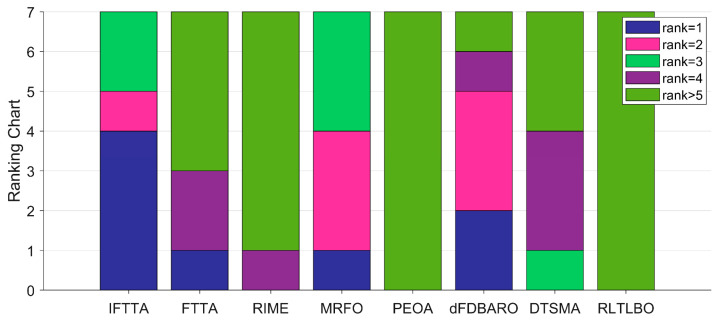
Box plots of IFTTA and competitors on eight functions.

In this section, three engineering-constrained optimization problems are introduced to verify the real-world problem-solving ability of the IFTTA. The specifics of the engineering-constrained optimization problem are shown in Table 12, where *D* represents the dimension of the problem, *g* is the number of inequality constraints, and *h* is the number of equality constraints. Table 13 records the mean, best value, standard deviation, and rank of the engineering problems solved by the IFTTA and the competitors. Figure 10 illustrates the ranking of each algorithm. The results show that the IFTTA performs best on four engineering problems, second on one problem, and third on two problems, which confirms the feasibility of the IFTTA on real optimization problems.

## 6. Conclusions

This paper proposes an improved version of the FTTA, called the IFTTA, to address the shortcomings of the FTTA by designing a fitness distance-balanced collective strategy, a non-monopoly extra training strategy, and a population restart strategy. In this paper, the performance of the IFTTA is confirmed by comparing it with three basic algorithms and three improved algorithms using the CEC2017 test suite (Dim = 10/30/50/100). The experimental results show that the IFTTA has better convergence accuracy and the ability to get rid of local optima compared to its competitors. The significant difference between the IFTTA and other algorithms was confirmed by using the Wilcoxon rank sum test, Friedman test, Iman–Davenport test, and Nemenyi test to statistically analyze the differences between these algorithms. Moreover, several engineering-constrained optimization problems confirm the potential of the IFTTA to solve real-world optimization problems. In conclusion, the IFTTA is an excellent human-based metaheuristic algorithm.

Despite the fact that the IFTTA has been proven to have better performance, it still performs poorly on some functions. Therefore, the structure of the IFTTA will be further optimized in the future. In addition, real-world optimization problems are more multi-objective with complex constraints, so it is our focus to present a multi-objective version and a constrained version of the IFTTA. Meanwhile, the IFTTA can be used to solve the UAV mission planning problem, the wireless sensor network coverage problem, the image segmentation problem, and so on.

## Figures and Tables

**Table 1:** Nine FTTA-NTS variants using different incorporation methods.

Algorithm	Cauchy		Gaussian	
	Equation (16)	Equation (17)	Equation (16)	Equation (17)
FTTA-NTS-1	×	×	×	√
FTTA-NTS-2	×	×	√	×
FTTA-NTS-3	×	√	×	×
FTTA-NTS-4	√	×	×	×
FTTA-NTS-5	×	×	√	√
FTTA-NTS-6	×	√	√	×
FTTA-NTS-7	×	×	√	√
FTTA-NTS-8	√	×	×	√
FTTA-NTS-9	×	×	×	×

**Table 2:** Parameter settings of each algorithm.

Algorithm	Parameter Setting
IFTTA	stu=0.3, com=0.3
FTTA	stu=0.3, com=0.3
RIME [40]	w=0.5
MRFO [41]	S=2
PEOA [42]	RE=0.5
dFDBARO [43]	k=1
DTSMA [44]	z=0.03, q=0.9
RLTLBO [45]	u=0.6, s=1, d=2, sgm=0.6, lbd=0.5

**Table 3:** The details of CEC 2017 test suite.

Type	ID	CEC2017 Function Name	Rang	Dimension	*f*_min_
Unimodal	F1	Shifted and Rotated Bent Cigar Function	[−100, 100]	10/30/50/100	100
	F2	Shifted and Rotated Zakharov Function	[−100, 100]	10/30/50/100	300
Multimodal	F3	Shifted and Rotated Rosenbrock’s Function	[−100, 100]	10/30/50/100	400
	F4	Shifted and Rotated Rastrigin’s Function	[−100, 100]	10/30/50/100	500
	F5	Shifted and Rotated Expanded Scaffer’s F6 Function	[−100, 100]	10/30/50/100	600
	F6	Shifted and Rotated Lunacek Bi_Rastrigin Function	[−100, 100]	10/30/50/100	700
	F7	Shifted and Rotated Non-Continuous Rastrigin’s Function	[−100, 100]	10/30/50/100	800
	F8	Shifted and Rotated Levy Function	[−100, 100]	10/30/50/100	900
	F9	Shifted and Rotated Schwefel’s Function	[−100, 100]	10/30/50/100	1000
Hybrid	F10	Hybrid Function 1 (N = 3)	[−100, 100]	10/30/50/100	1100
	F11	Hybrid Function 2 (N = 3)	[−100, 100]	10/30/50/100	1200
	F12	Hybrid Function 3 (N = 3)	[−100, 100]	10/30/50/100	1300
	F13	Hybrid Function 4 (N = 4)	[−100, 100]	10/30/50/100	1400
	F14	Hybrid Function 5 (N = 4)	[−100, 100]	10/30/50/100	1500
	F15	Hybrid Function 6 (N = 4)	[−100, 100]	10/30/50/100	1600
	F16	Hybrid Function 6 (N = 5)	[−100, 100]	10/30/50/100	1700
	F17	Hybrid Function 6 (N = 5)	[−100, 100]	10/30/50/100	1800
	F18	Hybrid Function 6 (N = 5)	[−100, 100]	10/30/50/100	1900
	F19	Hybrid Function 6 (N = 6)	[−100, 100]	10/30/50/100	2000
Composition	F20	Composition Function 1 (N = 3)	[−100, 100]	10/30/50/100	2100
	F21	Composition Function 2 (N = 3)	[−100, 100]	10/30/50/100	2200
	F22	Composition Function 3 (N = 4)	[−100, 100]	10/30/50/100	2300
	F23	Composition Function 4 (N = 4)	[−100, 100]	10/30/50/100	2400
	F24	Composition Function 5 (N = 5)	[−100, 100]	10/30/50/100	2500
	F25	Composition Function 6 (N = 5)	[−100, 100]	10/30/50/100	2600
	F26	Composition Function 7 (N = 6)	[−100, 100]	10/30/50/100	2700
	F27	Composition Function 8 (N = 6)	[−100, 100]	10/30/50/100	2800
	F28	Composition Function 9 (N = 3)	[−100, 100]	10/30/50/100	2900
	F29	Composition Function 10 (N = 3)	[−100, 100]	10/30/50/100	3000

**Table 4:** The rankings of FTTA and FTTA-FTS variants according to the Friedman test.

Test Suite	Dim	FTTA	FTTA-RFDB	FTTA-AFDB	FTTA-CFDB	FTTA-GFDB	Friedman *p*-Value
CEC2017	10	4.55	2.29	2.78	2.53	2.84	1.26 × 10^−7^
	30	4.38	1.48	3.55	2.55	3.03	3.85 × 10^−11^
	50	4.48	1.79	3.00	2.72	3.00	9.04 × 10^−9^
	100	4.79	1.28	3.66	2.21	3.07	2.34 × 10^−17^
Average rank		4.55	1.71	3.25	2.50	2.99	N/A

**Table 5:** The results of Wilcoxon rank sum test between FTTA and FTTA-FTS variants.

vs. FTTA +/=/−	CEC2017 with Dim = 10/30/50/100			
	10	30	50	100
FTTA-RFDB	17/9/3	22/6/1	21/6/2	24/5/0
FTTA-AFDB	20/8/1	16/11/2	18/11/0	14/15/0
FTTA-CFDB	20/8/1	17/11/1	18/10/1	24/5/0
FTTA-GFDB	21/7/1	17/10/2	19/10/0	23/6/0

**Table 6:** The rankings of FTTA and FTTA-NTS variants according to the Friedman test.

Test Suite	Dimension	FTTA	FTTA-NTS-1	FTTA-NTS-2	FTTA-NTS-3	FTTA-NTS-4	FTTA-NTS-5	FTTA-NTS-6	FTTA-NTS-7	FTTA-NTS-8	FTTA-NTS-9	Friedman *p*-Value
CEC 2017	10	5.74	5.12	5.86	5.03	5.76	5.28	5.66	5.64	4.84	6.07	8.64 × 10^−1^
	30	5.03	4.90	6.38	4.69	6.72	4.97	5.31	5.52	4.90	6.59	4.86 × 10^−2^
	50	6.83	4.83	6.62	5.03	6.24	4.38	4.52	4.86	4.24	7.45	1.28 × 10^−5^
	100	8.24	4.93	6.28	5.38	6.76	2.97	3.66	4.48	3.45	8.86	1.28 × 10^−20^
Average rank		6.46	4.94	6.28	5.03	6.37	4.40	4.78	5.13	4.36	7.24	N/A

**Table 7:** The results of Wilcoxon rank sum test between FTTA and FTTA-NTS variants.

vs. FTTA +/=/−	CEC2017 with Dim = 10/30/50/100			
	10	30	50	100
FTTA-NTS-1	3/23/3	4/17/8	11/14/4	20/7/2
FTTA-NTS-2	4/21/4	3/17/9	6/20/3	17/10/2
FTTA-NTS-3	5/21/3	5/16/8	11/14/4	20/7/2
FTTA-NTS-4	2/27/0	3/16/10	8/20/1	18/10/1
FTTA-NTS-5	3/22/4	4/16/9	10/16/3	22/6/1
FTTA-NTS-6	5/22/2	3/19/7	10/16/3	22/6/1
FTTA-NTS-7	4/25/0	3/16/10	10/18/1	19/8/2
FTTA-NTS-8	4/25/0	3/18/8	11/17/1	19/9/1
FTTA-NTS-9	3/24/2	4/15/10	8/17/4	12/14/3

**Table 8:** The rankings of FTTA and IFTTA variants according to the Friedman test.

Test Suite	Dimension	FTTA	FTTA-FTS	FTTA-NTS	FTTA-PRS	IFTTA	Friedman *p*-Value
CEC2017	10	4.36	2.52	3.98	2.69	1.45	2.53 × 10^−13^
	30	4.17	1.83	4.10	2.90	2.00	8.66 × 10^−12^
	50	4.38	2.14	3.69	2.97	1.83	1.27 × 10^−10^
	100	4.72	2.07	3.14	3.28	1.79	8.49 × 10^−13^
Average rank		4.41	2.14	3.73	2.96	1.77	N/A

**Table 9:** The results of Wilcoxon rank sum test between FTTA and IFTTA variants.

vs. FTTA +/=/−	CEC2017 with Dim = 10/30/50/100				
	10	30	50	100	Total
FTTA-FTS	17/9/3	22/6/1	21/6/2	24/5/0	84/26/6
FTTA-NTS	4/25/0	3/18/8	11/17/1	19/9/1	37/69/10
FTTA-PRS	14/14/1	9/20/0	14/15/0	21/8/0	58/57/1
IFTTA	27/2/0	22/6/1	22/6/1	26/3/0	87/17/2

**Table 10:** The results of Wilcoxon rank sum test between FTTA and competitors.

+/=/− IFTTA vs.	CEC2017 Test Suite			
	Dim = 10	Dim = 30	Dim = 50	Dim = 100
FTTA	27/2/0	22/6/1	22/6/1	26/3/0
RIME	24/2/3	25/3/1	26/2/1	28/1/0
MRFO	18/8/3	17/9/3	18/8/3	20/8/1
POEA	23/4/2	23/2/4	25/1/3	25/2/2
dFDBARO	12/8/9	20/5/4	18/10/1	22/4/3
DTSMA	20/5/4	23/4/2	25/3/1	27/1/1
RLTLBO	18/8/3	18/8/3	20/5/4	26/1/2

**Table 11:** The results of Wilcoxon rank sum test between FTTA and competitors.

Test Suite	Dimension	IFTTA	FTTA	RIME	MRFO	POEA	dFDBARO	DTSMA	RLTLBO	Friedman *p*-Value
CEC2017	10	2.69	6.07	5.62	5.07	6.10	2.41	4.41	3.62	4.14 × 10^−13^
	30	2.24	4.76	5.28	4.86	6.66	2.93	4.93	4.34	2.52 × 10^−11^
	50	2.03	4.24	5.38	4.45	6.62	3.31	4.66	5.31	1.30 × 10^−11^
	100	1.62	3.93	5.28	3.66	6.17	3.97	5.10	6.28	1.45 × 10^−14^
Mean rank		2.15	4.75	5.39	4.51	6.39	3.16	4.78	4.89	N/A

**Table 12:** Engineering-constrained optimization problems and features.

Problems	Name	*D*	*g*	*h*
EC1	Welded beam design	4	5	0
EC2	Pressure vessel design	4	4	0
EC3	Tension/compression spring design (case 1)	3	3	0
EC4	Gear train design Problem	4	1	1
EC5	Three-bar truss design problem	2	3	0
EC6	Cantilever beam design	5	1	0
EC7	Weight Minimization of a Speed Reducer	7	11	0

**Table 13:** The results of constrained optimization problem between IFTTA and competitors.

No.	Index	IFTTA	FTTA	RIME	MRFO	POEA	dFDBARO	DTSMA	RLTLBO
EC1	Best	1.724852	1.724852	1.743662	1.724852	1.737015	1.724852	1.724865	1.975659
	Ave	1.724852	1.749665	2.371319	1.724877	1.959929	1.724855	1.732449	2.503435
	Std	1.23 × 10^−14^	0.076744	0.525167	7.13 × 10^−5^	0.198885	7.20 × 10^−6^	0.02822	0.227793
	Rank	1	5	7	3	6	2	4	8
EC2	Best	5885.333	5885.88	6027.581	5890.976	5888.571	5885.356	5885.345	8007.555
	Ave	5971.134	6377.118	6777.524	5945.544	6758.23	5891.672	18590.68	38450.22
	Std	218.3567	492.4652	429.8811	48.40798	447.7315	19.1683	28752.09	47027.59
	Rank	3	4	6	2	5	1	7	8
EC3	Best	0.012665	0.012665	0.012755	0.012668	0.012676	0.012665	0.012665	0.012692
	Ave	0.012729	0.013076	0.016326	0.012747	0.013396	0.012676	0.014009	2326.591
	Std	0.000122	0.000867	0.002004	5.44 × 10^−5^	0.00079	2.95 × 10^−5^	0.001306	8902.235
	Rank	2	4	7	3	5	1	6	8
EC4	Best	2.70 × 10^−12^	1.17 × 10^−10^	2.70 × 10^−12^	2.70 × 10^−12^	2.70 × 10^−12^	2.70 × 10^−12^	2.31 × 10^−11^	1.55 × 10^−10^
	Ave	7.76 × 10^−10^	6.18 × 10^−9^	1.75 × 10^−9^	1.94 × 10^−10^	3.95 × 10^−9^	6.88 × 10^−10^	7.40 × 10^−9^	2.96 × 10^−7^
	Std	8.52 × 10^−10^	8.03 × 10^−9^	3.85 × 10^−9^	5.18 × 10^−10^	5.71 × 10^−9^	1.15 × 10^−9^	1.28 × 10^−8^	6.04 × 10^−7^
	Rank	3	6	4	1	5	2	7	8
EC5	Best	263.4634	263.4634	263.4661	263.4634	263.4634	263.4634	263.4634	263.4634
	Ave	263.4634	263.4635	264.1753	263.4634	263.4634	263.4634	263.4634	263.4785
	Std	3.22 × 10^−14^	0.000472	1.533939	6.68 × 10^−14^	4.03 × 10^−7^	6.23 × 10^−14^	4.43 × 10^−9^	0.04223
	Rank	1	6	8	2	5	2	4	7
EC6	Best	13.03252	13.03259	13.04384	13.03253	13.04794	13.03253	13.03253	13.14157
	Ave	13.03258	13.04086	13.3142	13.03259	13.11439	13.03291	13.03273	13.91878
	Std	6.48 × 10^−5^	0.034553	0.262858	6.22 × 10^−5^	0.053727	0.000553	0.000179	0.456248
	Rank	1	5	7	2	6	4	3	8
EC7	Best	2919.314	2919.314	2919.317	2919.314	2919.315	2919.314	2919.314	2933.098
	Ave	2919.314	2919.314	2919.401	2919.314	2921.788	2919.336	2919.314	3097.369
	Std	2.80 × 10^−13^	3.69 × 10^−13^	0.103366	4.35 × 10^−10^	2.986103	0.148957	5.29 × 10^−9^	102.5708
	Rank	1	1	6	3	7	5	4	8

## Data Availability

The data presented in this study are available upon request from the corresponding author.

## Appendix A

**Table A1:** Comparison of statistical results derived from IFTTA and competitors for CEC 2017 (Dim = 10).

Function	Index	IFTTA	FTTA	RIME	MRFO	POEA	dFDBARO	DTSMA	RLTLBO
F1	Best	1.00 × 10^2^	1.00 × 10^2^	3.85 × 10^2^	1.00 × 10^2^	1.01 × 10^2^	1.00 × 10^2^	1.15 × 10^2^	1.00 × 10^2^
	Ave	2.26 × 10^3^	2.89 × 10^3^	5.76 × 10^3^	1.64 × 10^3^	2.47 × 10^3^	1.00 × 10^2^	6.19 × 10^3^	2.48 × 10^3^
	Std	2.76 × 10^3^	3.24 × 10^3^	4.08 × 10^3^	1.61 × 10^3^	2.77 × 10^3^	5.95 × 10^−2^	4.12 × 10^3^	2.83 × 10^3^
	Rank	3	6	7	2	4	1	8	5
F2	Best	3.00 × 10^2^	3.00 × 10^2^	3.00 × 10^2^	3.00 × 10^2^	3.00 × 10^2^	3.00 × 10^2^	3.00 × 10^2^	3.00 × 10^2^
	Ave	3.00 × 10^2^	3.00 × 10^2^	3.00 × 10^2^	3.00 × 10^2^	3.00 × 10^2^	3.00 × 10^2^	3.00 × 10^2^	3.00 × 10^2^
	Std	1.61 × 10^−14^	8.95 × 10^−14^	5.25 × 10^−2^	2.54 × 10^−14^	1.36 × 10^−6^	1.92 × 10^−7^	2.88 × 10^−5^	1.04 × 10^−13^
	Rank	1	4	8	1	6	5	7	1
F3	Best	4.00 × 10^2^	4.00 × 10^2^	4.00 × 10^2^	4.00 × 10^2^	4.00 × 10^2^	4.00 × 10^2^	4.00 × 10^2^	4.00 × 10^2^
	Ave	4.00 × 10^2^	4.00 × 10^2^	4.08 × 10^2^	4.00 × 10^2^	4.06 × 10^2^	4.00 × 10^2^	4.03 × 10^2^	4.03 × 10^2^
	Std	1.44 × 10^−2^	3.54 × 10^−1^	1.35 × 10^1^	5.49 × 10^−2^	9.41 × 10^0^	3.63 × 10^−1^	7.55 × 10^−1^	1.36 × 10^0^
	Rank	1	3	8	2	7	4	6	5
F4	Best	5.02 × 10^2^	5.07 × 10^2^	5.02 × 10^2^	5.04 × 10^2^	5.08 × 10^2^	5.02 × 10^2^	5.02 × 10^2^	5.03 × 10^2^
	Ave	5.08 × 10^2^	5.15 × 10^2^	5.11 × 10^2^	5.19 × 10^2^	5.26 × 10^2^	5.11 × 10^2^	5.13 × 10^2^	5.11 × 10^2^
	Std	3.46 × 10^0^	5.78 × 10^0^	4.60 × 10^0^	8.77 × 10^0^	8.98 × 10^0^	4.93 × 10^0^	5.89 × 10^0^	4.59 × 10^0^
	Rank	1	6	4	7	8	3	5	2
F5	Best	6.00 × 10^2^	6.00 × 10^2^	6.00 × 10^2^	6.00 × 10^2^	6.01 × 10^2^	6.00 × 10^2^	6.00 × 10^2^	6.00 × 10^2^
	Ave	6.00 × 10^2^	6.00 × 10^2^	6.00 × 10^2^	6.00 × 10^2^	6.07 × 10^2^	6.00 × 10^2^	6.00 × 10^2^	6.00 × 10^2^
	Std	6.34 × 10^−5^	6.43 × 10^−2^	3.62 × 10^−2^	1.02 × 10^0^	3.65 × 10^0^	1.35 × 10^−3^	5.36 × 10^−2^	3.02 × 10^−1^
	Rank	1	3	5	7	8	2	4	6
F6	Best	7.12 × 10^2^	7.16 × 10^2^	7.10 × 10^2^	7.18 × 10^2^	7.18 × 10^2^	7.13 × 10^2^	7.10 × 10^2^	7.14 × 10^2^
	Ave	7.18 × 10^2^	7.27 × 10^2^	7.23 × 10^2^	7.39 × 10^2^	7.36 × 10^2^	7.22 × 10^2^	7.24 × 10^2^	7.24 × 10^2^
	Std	4.68 × 10^0^	9.07 × 10^0^	5.70 × 10^0^	1.47 × 10^1^	1.22 × 10^1^	5.56 × 10^0^	6.42 × 10^0^	6.98 × 10^0^
	Rank	1	6	3	8	7	2	5	4
F7	Best	8.02 × 10^2^	8.07 × 10^2^	8.04 × 10^2^	8.09 × 10^2^	8.08 × 10^2^	8.03 × 10^2^	8.03 × 10^2^	8.05 × 10^2^
	Ave	8.08 × 10^2^	8.15 × 10^2^	8.12 × 10^2^	8.22 × 10^2^	8.20 × 10^2^	8.12 × 10^2^	8.15 × 10^2^	8.14 × 10^2^
	Std	4.17 × 10^0^	5.68 × 10^0^	6.08 × 10^0^	8.35 × 10^0^	7.72 × 10^0^	5.00 × 10^0^	6.77 × 10^0^	5.05 × 10^0^
	Rank	1	6	3	8	7	2	5	4
F8	Best	9.00 × 10^2^	9.00 × 10^2^	9.00 × 10^2^	9.00 × 10^2^	9.00 × 10^2^	9.00 × 10^2^	9.00 × 10^2^	9.00 × 10^2^
	Ave	9.00 × 10^2^	9.05 × 10^2^	9.00 × 10^2^	9.01 × 10^2^	9.05 × 10^2^	9.00 × 10^2^	9.00 × 10^2^	9.00 × 10^2^
	Std	0.00 × 10^0^	1.02 × 10^1^	4.92 × 10^−1^	3.54 × 10^0^	1.22 × 10^1^	1.41 × 10^−1^	1.23 × 10^−1^	6.83 × 10^−1^
	Rank	1	7	4	6	8	2	3	5
F9	Best	1.01 × 10^3^	1.02 × 10^3^	1.01 × 10^3^	1.12 × 10^3^	1.22 × 10^3^	1.00 × 10^3^	1.15 × 10^3^	1.03 × 10^3^
	Ave	1.42 × 10^3^	1.59 × 10^3^	1.33 × 10^3^	1.65 × 10^3^	1.67 × 10^3^	1.37 × 10^3^	1.40 × 10^3^	1.40 × 10^3^
	Std	2.29 × 10^2^	2.91 × 10^2^	1.72 × 10^2^	2.79 × 10^2^	2.10 × 10^2^	2.00 × 10^2^	1.13 × 10^2^	2.29 × 10^2^
	Rank	5	6	1	7	8	2	3	4
F10	Best	1.10 × 10^3^	1.10 × 10^3^	1.10 × 10^3^	1.10 × 10^3^	1.11 × 10^3^	1.10 × 10^3^	1.10 × 10^3^	1.10 × 10^3^
	Ave	1.10 × 10^3^	1.11 × 10^3^	1.11 × 10^3^	1.11 × 10^3^	1.14 × 10^3^	1.10 × 10^3^	1.11 × 10^3^	1.11 × 10^3^
	Std	1.57 × 10^0^	8.22 × 10^0^	5.63 × 10^0^	8.68 × 10^0^	1.76 × 10^1^	2.89 × 10^0^	6.32 × 10^0^	5.93 × 10^0^
	Rank	1	7	6	5	8	2	3	4
F11	Best	1.53 × 10^3^	1.31 × 10^3^	2.00 × 10^3^	1.46 × 10^3^	4.29 × 10^3^	1.20 × 10^3^	1.72 × 10^3^	1.78 × 10^3^
	Ave	1.51 × 10^4^	1.52 × 10^4^	2.05 × 10^4^	1.16 × 10^4^	5.81 × 10^4^	3.77 × 10^3^	2.19 × 10^4^	1.07 × 10^4^
	Std	1.59 × 10^4^	1.50 × 10^4^	1.57 × 10^4^	1.19 × 10^4^	7.11 × 10^4^	5.94 × 10^3^	2.05 × 10^4^	7.16 × 10^3^
	Rank	4	5	6	3	8	1	7	2
F12	Best	1.31 × 10^3^	1.31 × 10^3^	1.34 × 10^3^	1.33 × 10^3^	1.86 × 10^3^	1.30 × 10^3^	1.33 × 10^3^	1.32 × 10^3^
	Ave	2.26 × 10^3^	7.99 × 10^3^	1.14 × 10^4^	2.09 × 10^3^	9.98 × 10^3^	1.31 × 10^3^	1.08 × 10^4^	2.74 × 10^3^
	Std	2.53 × 10^3^	7.06 × 10^3^	1.03 × 10^4^	7.02 × 10^2^	5.45 × 10^3^	4.36 × 10^0^	1.11 × 10^4^	1.84 × 10^3^
	Rank	3	5	8	2	6	1	7	4
F13	Best	1.40 × 10^3^	1.41 × 10^3^	1.40 × 10^3^	1.43 × 10^3^	1.43 × 10^3^	1.40 × 10^3^	1.40 × 10^3^	1.41 × 10^3^
	Ave	1.42 × 10^3^	1.53 × 10^3^	1.98 × 10^3^	1.45 × 10^3^	1.46 × 10^3^	1.40 × 10^3^	1.44 × 10^3^	1.43 × 10^3^
	Std	2.93 × 10^1^	2.16 × 10^2^	1.26 × 10^3^	1.19 × 10^1^	2.87 × 10^1^	3.03 × 10^0^	1.13 × 10^2^	1.00 × 10^1^
	Rank	2	7	8	5	6	1	4	3
F14	Best	1.50 × 10^3^	1.50 × 10^3^	1.50 × 10^3^	1.51 × 10^3^	1.52 × 10^3^	1.50 × 10^3^	1.50 × 10^3^	1.51 × 10^3^
	Ave	1.51 × 10^3^	1.55 × 10^3^	2.73 × 10^3^	1.56 × 10^3^	1.64 × 10^3^	1.50 × 10^3^	1.55 × 10^3^	1.53 × 10^3^
	Std	1.52 × 10^1^	9.08 × 10^1^	2.01 × 10^3^	3.40 × 10^1^	8.05 × 10^1^	1.67 × 10^0^	9.07 × 10^1^	2.00 × 10^1^
	Rank	2	4	8	6	7	1	5	3
F15	Best	1.60 × 10^3^	1.60 × 10^3^	1.60 × 10^3^	1.60 × 10^3^	1.60 × 10^3^	1.60 × 10^3^	1.60 × 10^3^	1.60 × 10^3^
	Ave	1.62 × 10^3^	1.70 × 10^3^	1.71 × 10^3^	1.71 × 10^3^	1.67 × 10^3^	1.66 × 10^3^	1.65 × 10^3^	1.62 × 10^3^
	Std	3.14 × 10^1^	9.44 × 10^1^	1.20 × 10^2^	1.16 × 10^2^	8.18 × 10^1^	8.48 × 10^1^	5.11 × 10^1^	4.26 × 10^1^
	Rank	1	6	8	7	5	4	3	2
F16	Best	1.70 × 10^3^	1.70 × 10^3^	1.70 × 10^3^	1.70 × 10^3^	1.73 × 10^3^	1.70 × 10^3^	1.70 × 10^3^	1.71 × 10^3^
	Ave	1.71 × 10^3^	1.73 × 10^3^	1.75 × 10^3^	1.74 × 10^3^	1.75 × 10^3^	1.71 × 10^3^	1.73 × 10^3^	1.74 × 10^3^
	Std	1.25 × 10^1^	2.79 × 10^1^	4.88 × 10^1^	3.09 × 10^1^	1.32 × 10^1^	1.28 × 10^1^	1.15 × 10^1^	1.00 × 10^1^
	Rank	2	4	7	5	8	1	3	6
F17	Best	1.80 × 10^3^	1.81 × 10^3^	1.91 × 10^3^	1.87 × 10^3^	2.15 × 10^3^	1.80 × 10^3^	2.09 × 10^3^	2.01 × 10^3^
	Ave	5.09 × 10^3^	5.53 × 10^3^	1.05 × 10^4^	5.00 × 10^3^	1.49 × 10^4^	1.80 × 10^3^	2.23 × 10^4^	4.20 × 10^3^
	Std	5.63 × 10^3^	8.14 × 10^3^	7.14 × 10^3^	3.35 × 10^3^	9.82 × 10^3^	1.80 × 10^0^	1.37 × 10^4^	2.47 × 10^3^
	Rank	4	5	6	3	7	1	8	2
F18	Best	1.90 × 10^3^	1.90 × 10^3^	1.90 × 10^3^	1.91 × 10^3^	1.91 × 10^3^	1.90 × 10^3^	1.90 × 10^3^	1.91 × 10^3^
	Ave	1.90 × 10^3^	3.38 × 10^3^	2.73 × 10^3^	1.96 × 10^3^	2.03 × 10^3^	1.90 × 10^3^	1.96 × 10^3^	1.92 × 10^3^
	Std	3.10 × 10^0^	5.26 × 10^3^	1.52 × 10^3^	5.66 × 10^1^	2.03 × 10^2^	6.64 × 10^−1^	1.17 × 10^2^	1.25 × 10^1^
	Rank	2	8	7	5	6	1	4	3
F19	Best	2.00 × 10^3^	2.00 × 10^3^	2.00 × 10^3^	2.00 × 10^3^	2.03 × 10^3^	2.00 × 10^3^	2.00 × 10^3^	2.00 × 10^3^
	Ave	2.00 × 10^3^	2.02 × 10^3^	2.02 × 10^3^	2.03 × 10^3^	2.05 × 10^3^	2.01 × 10^3^	2.01 × 10^3^	2.02 × 10^3^
	Std	3.09 × 10^0^	2.50 × 10^1^	3.00 × 10^1^	3.79 × 10^1^	1.48 × 10^1^	8.37 × 10^0^	9.95 × 10^0^	7.85 × 10^0^
	Rank	1	5	4	7	8	2	3	6
F20	Best	2.20 × 10^3^	2.20 × 10^3^	2.20 × 10^3^	2.20 × 10^3^	2.20 × 10^3^	2.20 × 10^3^	2.20 × 10^3^	2.20 × 10^3^
	Ave	2.29 × 10^3^	2.30 × 10^3^	2.29 × 10^3^	2.22 × 10^3^	2.20 × 10^3^	2.26 × 10^3^	2.22 × 10^3^	2.22 × 10^3^
	Std	4.58 × 10^1^	4.55 × 10^1^	5.18 × 10^1^	4.13 × 10^1^	8.18 × 10^−1^	5.72 × 10^1^	4.60 × 10^1^	4.04 × 10^1^
	Rank	6	8	7	2	1	5	4	3
F21	Best	2.21 × 10^3^	2.22 × 10^3^	2.22 × 10^3^	2.25 × 10^3^	2.21 × 10^3^	2.22 × 10^3^	2.21 × 10^3^	2.23 × 10^3^
	Ave	2.29 × 10^3^	2.30 × 10^3^	2.29 × 10^3^	2.30 × 10^3^	2.30 × 10^3^	2.30 × 10^3^	2.30 × 10^3^	2.30 × 10^3^
	Std	2.26 × 10^1^	2.19 × 10^1^	2.39 × 10^1^	7.87 × 10^0^	2.55 × 10^1^	1.76 × 10^1^	2.32 × 10^1^	1.12 × 10^1^
	Rank	1	4	2	8	7	5	3	6
F22	Best	2.60 × 10^3^	2.61 × 10^3^	2.61 × 10^3^	2.61 × 10^3^	2.60 × 10^3^	2.61 × 10^3^	2.61 × 10^3^	2.60 × 10^3^
	Ave	2.61 × 10^3^	2.62 × 10^3^	2.62 × 10^3^	2.62 × 10^3^	2.63 × 10^3^	2.61 × 10^3^	2.62 × 10^3^	2.61 × 10^3^
	Std	5.00 × 10^0^	9.45 × 10^0^	5.30 × 10^0^	1.13 × 10^1^	9.78 × 10^0^	5.86 × 10^0^	5.83 × 10^0^	5.29 × 10^0^
	Rank	1	7	4	6	8	3	5	2
F23	Best	2.50 × 10^3^	2.50 × 10^3^	2.50 × 10^3^	2.50 × 10^3^	2.50 × 10^3^	2.50 × 10^3^	2.50 × 10^3^	2.50 × 10^3^
	Ave	2.73 × 10^3^	2.74 × 10^3^	2.73 × 10^3^	2.66 × 10^3^	2.51 × 10^3^	2.68 × 10^3^	2.72 × 10^3^	2.69 × 10^3^
	Std	4.72 × 10^1^	5.02 × 10^1^	6.72 × 10^1^	1.18 × 10^2^	3.36 × 10^1^	1.07 × 10^2^	8.71 × 10^1^	9.47 × 10^1^
	Rank	7	8	6	2	1	3	5	4
F24	Best	2.90 × 10^3^	2.90 × 10^3^	2.90 × 10^3^	2.90 × 10^3^	2.90 × 10^3^	2.90 × 10^3^	2.90 × 10^3^	2.90 × 10^3^
	Ave	2.93 × 10^3^	2.93 × 10^3^	2.93 × 10^3^	2.93 × 10^3^	2.92 × 10^3^	2.92 × 10^3^	2.92 × 10^3^	2.92 × 10^3^
	Std	2.28 × 10^1^	2.26 × 10^1^	2.48 × 10^1^	2.29 × 10^1^	2.31 × 10^1^	2.31 × 10^1^	2.61 × 10^1^	2.28 × 10^1^
	Rank	7	8	5	6	4	3	2	1
F25	Best	2.80 × 10^3^	2.60 × 10^3^	2.60 × 10^3^	2.60 × 10^3^	2.60 × 10^3^	2.90 × 10^3^	2.90 × 10^3^	2.80 × 10^3^
	Ave	2.93 × 10^3^	3.04 × 10^3^	2.94 × 10^3^	2.90 × 10^3^	2.89 × 10^3^	2.90 × 10^3^	2.93 × 10^3^	2.93 × 10^3^
	Std	4.29 × 10^1^	2.70 × 10^2^	1.94 × 10^2^	1.17 × 10^2^	5.33 × 10^1^	6.60 × 10^0^	3.78 × 10^1^	8.10 × 10^1^
	Rank	5	8	7	2	1	3	4	6
F26	Best	3.09 × 10^3^	3.09 × 10^3^	3.09 × 10^3^	3.09 × 10^3^	3.09 × 10^3^	3.09 × 10^3^	3.09 × 10^3^	3.09 × 10^3^
	Ave	3.09 × 10^3^	3.10 × 10^3^	3.09 × 10^3^	3.11 × 10^3^	3.09 × 10^3^	3.09 × 10^3^	3.09 × 10^3^	3.09 × 10^3^
	Std	1.90 × 10^0^	8.70 × 10^0^	7.51 × 10^0^	1.65 × 10^1^	3.23 × 10^0^	2.70 × 10^0^	2.12 × 10^0^	3.37 × 10^0^
	Rank	2	7	5	8	6	4	1	3
F27	Best	3.10 × 10^3^	3.10 × 10^3^	2.80 × 10^3^	2.80 × 10^3^	3.10 × 10^3^	3.10 × 10^3^	3.10 × 10^3^	3.10 × 10^3^
	Ave	3.26 × 10^3^	3.33 × 10^3^	3.26 × 10^3^	3.21 × 10^3^	3.13 × 10^3^	3.13 × 10^3^	3.23 × 10^3^	3.19 × 10^3^
	Std	1.48 × 10^2^	1.26 × 10^2^	1.48 × 10^2^	1.52 × 10^2^	7.49 × 10^1^	8.34 × 10^1^	1.46 × 10^2^	1.15 × 10^2^
	Rank	7	8	6	4	2	1	5	3
F28	Best	3.13 × 10^3^	3.14 × 10^3^	3.14 × 10^3^	3.14 × 10^3^	3.14 × 10^3^	3.14 × 10^3^	3.13 × 10^3^	3.14 × 10^3^
	Ave	3.16 × 10^3^	3.20 × 10^3^	3.18 × 10^3^	3.20 × 10^3^	3.20 × 10^3^	3.16 × 10^3^	3.16 × 10^3^	3.16 × 10^3^
	Std	2.07 × 10^1^	5.19 × 10^1^	3.09 × 10^1^	3.79 × 10^1^	2.86 × 10^1^	2.18 × 10^1^	2.51 × 10^1^	1.23 × 10^1^
	Rank	1	8	5	6	7	3	2	4
F29	Best	3.53 × 10^3^	3.60 × 10^3^	4.60 × 10^3^	3.62 × 10^3^	4.94 × 10^3^	3.53 × 10^3^	3.94 × 10^3^	3.80 × 10^3^
	Ave	1.02 × 10^5^	3.51 × 10^5^	1.63 × 10^5^	1.95 × 10^5^	4.42 × 10^5^	3.69 × 10^4^	1.33 × 10^5^	2.01 × 10^4^
	Std	2.65 × 10^5^	4.02 × 10^5^	3.30 × 10^5^	4.19 × 10^5^	6.15 × 10^5^	1.60 × 10^5^	3.10 × 10^5^	3.95 × 10^4^
	Rank	3	7	5	6	8	2	4	1

**Table A2:** Comparison of statistical results derived from IFTTA and competitors for CEC 2017 (Dim = 30).

Function	Index	IFTTA	FTTA	RIME	MRFO	POEA	dFDBARO	DTSMA	RLTLBO
F1	Best	1.09 × 10^2^	1.04 × 10^2^	1.21 × 10^5^	1.03 × 10^2^	1.00 × 10^2^	9.94 × 10^2^	1.03 × 10^2^	1.68 × 10^2^
	Ave	4.20 × 10^3^	4.94 × 10^3^	3.94 × 10^5^	4.85 × 10^3^	2.69 × 10^3^	1.11 × 10^4^	6.58 × 10^3^	2.61 × 10^4^
	Std	4.72 × 10^3^	5.44 × 10^3^	1.81 × 10^5^	5.27 × 10^3^	3.65 × 10^3^	9.07 × 10^3^	7.41 × 10^3^	8.31 × 10^4^
	Rank	2	4	8	3	1	6	5	7
F2	Best	5.93 × 10^3^	3.55 × 10^3^	1.94 × 10^3^	4.42 × 10^2^	3.00 × 10^2^	8.36 × 10^3^	5.08 × 10^2^	1.25 × 10^3^
	Ave	1.65 × 10^4^	1.15 × 10^4^	5.94 × 10^3^	2.39 × 10^3^	3.00 × 10^2^	2.12 × 10^4^	4.74 × 10^3^	3.64 × 10^3^
	Std	6.64 × 10^3^	4.94 × 10^3^	2.33 × 10^3^	1.75 × 10^3^	2.05 × 10^−1^	6.25 × 10^3^	4.20 × 10^3^	1.97 × 10^3^
	Rank	7	6	5	2	1	8	4	3
F3	Best	4.01 × 10^2^	4.02 × 10^2^	4.44 × 10^2^	4.00 × 10^2^	4.69 × 10^2^	4.10 × 10^2^	4.24 × 10^2^	4.71 × 10^2^
	Ave	4.76 × 10^2^	4.85 × 10^2^	5.07 × 10^2^	4.71 × 10^2^	5.09 × 10^2^	4.94 × 10^2^	4.96 × 10^2^	5.08 × 10^2^
	Std	1.60 × 10^1^	3.00 × 10^1^	2.91 × 10^1^	3.09 × 10^1^	2.24 × 10^1^	2.70 × 10^1^	1.92 × 10^1^	2.73 × 10^1^
	Rank	2	3	6	1	8	4	5	7
F4	Best	5.24 × 10^2^	5.47 × 10^2^	5.44 × 10^2^	5.83 × 10^2^	5.99 × 10^2^	5.51 × 10^2^	5.45 × 10^2^	5.73 × 10^2^
	Ave	5.71 × 10^2^	6.03 × 10^2^	5.81 × 10^2^	6.53 × 10^2^	6.74 × 10^2^	5.86 × 10^2^	5.93 × 10^2^	6.19 × 10^2^
	Std	3.25 × 10^1^	3.00 × 10^1^	2.52 × 10^1^	4.03 × 10^1^	3.16 × 10^1^	2.16 × 10^1^	2.38 × 10^1^	2.24 × 10^1^
	Rank	1	5	2	7	8	3	4	6
F5	Best	6.00 × 10^2^	6.00 × 10^2^	6.01 × 10^2^	6.01 × 10^2^	6.24 × 10^2^	6.00 × 10^2^	6.01 × 10^2^	6.01 × 10^2^
	Ave	6.00 × 10^2^	6.01 × 10^2^	6.04 × 10^2^	6.15 × 10^2^	6.44 × 10^2^	6.01 × 10^2^	6.05 × 10^2^	6.12 × 10^2^
	Std	6.36 × 10^−3^	7.65 × 10^−1^	2.83 × 10^0^	1.09 × 10^1^	8.10 × 10^0^	1.50 × 10^0^	2.50 × 10^0^	5.51 × 10^0^
	Rank	1	2	4	7	8	3	5	6
F6	Best	7.59 × 10^2^	7.91 × 10^2^	7.72 × 10^2^	8.13 × 10^2^	8.77 × 10^2^	7.98 × 10^2^	7.85 × 10^2^	8.00 × 10^2^
	Ave	8.02 × 10^2^	8.67 × 10^2^	8.22 × 10^2^	9.41 × 10^2^	9.84 × 10^2^	8.42 × 10^2^	8.38 × 10^2^	8.77 × 10^2^
	Std	3.11 × 10^1^	3.46 × 10^1^	2.46 × 10^1^	6.05 × 10^1^	5.01 × 10^1^	2.70 × 10^1^	3.04 × 10^1^	4.12 × 10^1^
	Rank	1	5	2	7	8	4	3	6
F7	Best	8.26 × 10^2^	8.42 × 10^2^	8.47 × 10^2^	8.50 × 10^2^	8.78 × 10^2^	8.38 × 10^2^	8.55 × 10^2^	8.50 × 10^2^
	Ave	8.72 × 10^2^	8.89 × 10^2^	8.84 × 10^2^	9.34 × 10^2^	9.30 × 10^2^	8.80 × 10^2^	8.90 × 10^2^	9.03 × 10^2^
	Std	3.26 × 10^1^	2.57 × 10^1^	1.87 × 10^1^	3.63 × 10^1^	2.49 × 10^1^	2.13 × 10^1^	2.48 × 10^1^	2.77 × 10^1^
	Rank	1	4	3	8	7	2	5	6
F8	Best	9.01 × 10^2^	1.03 × 10^3^	9.24 × 10^2^	1.24 × 10^3^	2.23 × 10^3^	9.81 × 10^2^	1.00 × 10^3^	1.01 × 10^3^
	Ave	9.11 × 10^2^	1.75 × 10^3^	1.58 × 10^3^	2.85 × 10^3^	3.68 × 10^3^	1.66 × 10^3^	2.20 × 10^3^	1.82 × 10^3^
	Std	1.06 × 10^1^	4.34 × 10^2^	8.69 × 10^2^	1.03 × 10^3^	8.01 × 10^2^	5.08 × 10^2^	8.99 × 10^2^	7.32 × 10^2^
	Rank	1	4	2	7	8	3	6	5
F9	Best	2.47 × 10^3^	2.68 × 10^3^	2.75 × 10^3^	3.25 × 10^3^	3.91 × 10^3^	2.55 × 10^3^	3.50 × 10^3^	4.26 × 10^3^
	Ave	3.93 × 10^3^	4.17 × 10^3^	4.24 × 10^3^	4.73 × 10^3^	5.34 × 10^3^	3.87 × 10^3^	4.86 × 10^3^	5.57 × 10^3^
	Std	5.32 × 10^2^	4.81 × 10^2^	6.50 × 10^2^	6.55 × 10^2^	6.42 × 10^2^	5.48 × 10^2^	6.22 × 10^2^	5.03 × 10^2^
	Rank	2	3	4	5	7	1	6	8
F10	Best	1.11 × 10^3^	1.13 × 10^3^	1.21 × 10^3^	1.13 × 10^3^	1.16 × 10^3^	1.12 × 10^3^	1.14 × 10^3^	1.15 × 10^3^
	Ave	1.14 × 10^3^	1.21 × 10^3^	1.30 × 10^3^	1.17 × 10^3^	1.24 × 10^3^	1.16 × 10^3^	1.25 × 10^3^	1.23 × 10^3^
	Std	2.20 × 10^1^	4.54 × 10^1^	4.50 × 10^1^	3.30 × 10^1^	4.83 × 10^1^	3.33 × 10^1^	5.45 × 10^1^	4.80 × 10^1^
	Rank	1	4	8	3	6	2	7	5
F11	Best	1.03 × 10^4^	2.09 × 10^4^	2.33 × 10^5^	1.26 × 10^4^	1.99 × 10^5^	2.05 × 10^4^	5.09 × 10^4^	1.35 × 10^4^
	Ave	6.77 × 10^4^	2.15 × 10^5^	7.03 × 10^6^	1.10 × 10^5^	5.58 × 10^6^	2.98 × 10^5^	1.19 × 10^6^	2.12 × 10^5^
	Std	6.58 × 10^4^	2.51 × 10^5^	5.91 × 10^6^	8.50 × 10^4^	4.42 × 10^6^	2.96 × 10^5^	9.36 × 10^5^	2.76 × 10^5^
	Rank	1	4	8	2	7	5	6	3
F12	Best	1.39 × 10^3^	1.40 × 10^3^	1.19 × 10^4^	1.38 × 10^3^	1.87 × 10^4^	1.38 × 10^3^	1.46 × 10^3^	2.18 × 10^3^
	Ave	2.15 × 10^4^	1.94 × 10^4^	6.67 × 10^4^	1.70 × 10^4^	7.69 × 10^4^	1.38 × 10^4^	2.06 × 10^4^	1.23 × 10^4^
	Std	1.99 × 10^4^	1.95 × 10^4^	8.09 × 10^4^	1.73 × 10^4^	4.68 × 10^4^	1.29 × 10^4^	2.26 × 10^4^	9.48 × 10^3^
	Rank	6	4	7	3	8	2	5	1
F13	Best	1.51 × 10^3^	2.43 × 10^3^	4.55 × 10^3^	1.72 × 10^3^	1.84 × 10^3^	1.46 × 10^3^	2.82 × 10^3^	1.66 × 10^3^
	Ave	8.29 × 10^3^	3.98 × 10^4^	3.18 × 10^4^	4.38 × 10^3^	9.14 × 10^3^	3.54 × 10^3^	4.28 × 10^4^	4.38 × 10^3^
	Std	8.56 × 10^3^	4.72 × 10^4^	2.79 × 10^4^	3.48 × 10^3^	6.85 × 10^3^	2.84 × 10^3^	3.20 × 10^4^	2.89 × 10^3^
	Rank	4	7	6	3	5	1	8	2
F14	Best	1.52 × 10^3^	1.55 × 10^3^	2.39 × 10^3^	1.55 × 10^3^	8.20 × 10^3^	1.54 × 10^3^	1.56 × 10^3^	1.70 × 10^3^
	Ave	6.90 × 10^3^	1.19 × 10^4^	1.48 × 10^4^	7.52 × 10^3^	3.62 × 10^4^	6.62 × 10^3^	2.14 × 10^4^	4.82 × 10^3^
	Std	7.68 × 10^3^	1.25 × 10^4^	1.34 × 10^4^	7.82 × 10^3^	2.75 × 10^4^	6.82 × 10^3^	1.44 × 10^4^	2.82 × 10^3^
	Rank	3	5	6	4	8	2	7	1
F15	Best	1.84 × 10^3^	1.75 × 10^3^	1.72 × 10^3^	1.97 × 10^3^	2.17 × 10^3^	1.76 × 10^3^	1.78 × 10^3^	1.71 × 10^3^
	Ave	2.40 × 10^3^	2.57 × 10^3^	2.51 × 10^3^	2.54 × 10^3^	3.08 × 10^3^	2.46 × 10^3^	2.35 × 10^3^	2.35 × 10^3^
	Std	3.17 × 10^2^	3.08 × 10^2^	3.06 × 10^2^	2.55 × 10^2^	3.27 × 10^2^	2.74 × 10^2^	3.25 × 10^2^	2.93 × 10^2^
	Rank	3	7	5	6	8	4	1	2
F16	Best	1.72 × 10^3^	1.78 × 10^3^	1.80 × 10^3^	1.73 × 10^3^	1.83 × 10^3^	1.74 × 10^3^	1.80 × 10^3^	1.78 × 10^3^
	Ave	1.90 × 10^3^	2.19 × 10^3^	2.10 × 10^3^	2.13 × 10^3^	2.21 × 10^3^	2.02 × 10^3^	2.15 × 10^3^	1.94 × 10^3^
	Std	1.62 × 10^2^	2.20 × 10^2^	1.84 × 10^2^	2.02 × 10^2^	1.82 × 10^2^	1.67 × 10^2^	2.03 × 10^2^	1.28 × 10^2^
	Rank	1	7	4	5	8	3	6	2
F17	Best	1.16 × 10^4^	2.70 × 10^4^	9.98 × 10^4^	2.15 × 10^4^	3.89 × 10^4^	1.36 × 10^4^	3.31 × 10^4^	3.73 × 10^4^
	Ave	1.85 × 10^5^	1.50 × 10^5^	5.60 × 10^5^	1.68 × 10^5^	1.28 × 10^5^	8.87 × 10^4^	5.02 × 10^5^	1.36 × 10^5^
	Std	1.73 × 10^5^	1.13 × 10^5^	4.00 × 10^5^	1.54 × 10^5^	1.02 × 10^5^	1.09 × 10^5^	4.57 × 10^5^	7.11 × 10^4^
	Rank	6	4	8	5	2	1	7	3
F18	Best	2.04 × 10^3^	1.95 × 10^3^	2.06 × 10^3^	1.92 × 10^3^	2.54 × 10^3^	1.92 × 10^3^	1.93 × 10^3^	2.11 × 10^3^
	Ave	9.78 × 10^3^	1.25 × 10^4^	1.48 × 10^4^	8.47 × 10^3^	1.14 × 10^5^	6.12 × 10^3^	2.46 × 10^4^	5.84 × 10^3^
	Std	1.28 × 10^4^	1.43 × 10^4^	1.29 × 10^4^	8.11 × 10^3^	6.50 × 10^4^	6.13 × 10^3^	2.00 × 10^4^	3.83 × 10^3^
	Rank	4	5	6	3	8	2	7	1
F19	Best	2.01 × 10^3^	2.07 × 10^3^	2.05 × 10^3^	2.17 × 10^3^	2.18 × 10^3^	2.16 × 10^3^	2.06 × 10^3^	2.10 × 10^3^
	Ave	2.24 × 10^3^	2.40 × 10^3^	2.40 × 10^3^	2.44 × 10^3^	2.42 × 10^3^	2.38 × 10^3^	2.41 × 10^3^	2.32 × 10^3^
	Std	1.59 × 10^2^	1.71 × 10^2^	1.88 × 10^2^	1.62 × 10^2^	1.07 × 10^2^	1.51 × 10^2^	1.81 × 10^2^	6.52 × 10^1^
	Rank	1	4	5	8	7	3	6	2
F20	Best	2.32 × 10^3^	2.35 × 10^3^	2.34 × 10^3^	2.20 × 10^3^	2.40 × 10^3^	2.34 × 10^3^	2.34 × 10^3^	2.34 × 10^3^
	Ave	2.36 × 10^3^	2.39 × 10^3^	2.38 × 10^3^	2.40 × 10^3^	2.46 × 10^3^	2.37 × 10^3^	2.39 × 10^3^	2.38 × 10^3^
	Std	3.94 × 10^1^	2.29 × 10^1^	2.40 × 10^1^	4.28 × 10^1^	3.06 × 10^1^	2.29 × 10^1^	2.86 × 10^1^	1.81 × 10^1^
	Rank	1	5	4	7	8	2	6	3
F21	Best	2.30 × 10^3^	2.30 × 10^3^	2.30 × 10^3^	2.30 × 10^3^	2.31 × 10^3^	2.30 × 10^3^	2.30 × 10^3^	2.30 × 10^3^
	Ave	2.90 × 10^3^	4.81 × 10^3^	4.38 × 10^3^	2.39 × 10^3^	2.33 × 10^3^	2.30 × 10^3^	5.31 × 10^3^	2.31 × 10^3^
	Std	1.24 × 10^3^	1.80 × 10^3^	1.69 × 10^3^	6.47 × 10^2^	5.30 × 10^1^	1.48 × 10^0^	1.63 × 10^3^	8.62 × 10^0^
	Rank	5	7	6	4	3	1	8	2
F22	Best	2.66 × 10^3^	2.72 × 10^3^	2.70 × 10^3^	2.71 × 10^3^	2.77 × 10^3^	2.68 × 10^3^	2.70 × 10^3^	2.71 × 10^3^
	Ave	2.70 × 10^3^	2.75 × 10^3^	2.74 × 10^3^	2.80 × 10^3^	2.86 × 10^3^	2.74 × 10^3^	2.74 × 10^3^	2.77 × 10^3^
	Std	3.48 × 10^1^	2.59 × 10^1^	2.48 × 10^1^	5.10 × 10^1^	4.72 × 10^1^	2.48 × 10^1^	2.16 × 10^1^	4.01 × 10^1^
	Rank	1	5	4	7	8	3	2	6
F23	Best	2.83 × 10^3^	2.86 × 10^3^	2.87 × 10^3^	2.87 × 10^3^	2.91 × 10^3^	2.87 × 10^3^	2.87 × 10^3^	2.86 × 10^3^
	Ave	2.89 × 10^3^	2.93 × 10^3^	2.92 × 10^3^	2.96 × 10^3^	3.01 × 10^3^	2.91 × 10^3^	2.91 × 10^3^	2.92 × 10^3^
	Std	2.93 × 10^1^	3.20 × 10^1^	2.99 × 10^1^	4.91 × 10^1^	5.69 × 10^1^	3.08 × 10^1^	2.50 × 10^1^	2.92 × 10^1^
	Rank	1	6	4	7	8	2	3	5
F24	Best	2.88 × 10^3^	2.88 × 10^3^	2.89 × 10^3^	2.88 × 10^3^	2.90 × 10^3^	2.88 × 10^3^	2.88 × 10^3^	2.89 × 10^3^
	Ave	2.89 × 10^3^	2.90 × 10^3^	2.90 × 10^3^	2.89 × 10^3^	2.95 × 10^3^	2.89 × 10^3^	2.89 × 10^3^	2.92 × 10^3^
	Std	1.37 × 10^1^	1.82 × 10^1^	1.48 × 10^1^	1.59 × 10^1^	2.19 × 10^1^	1.31 × 10^1^	7.44 × 10^0^	2.79 × 10^1^
	Rank	2	6	5	4	8	3	1	7
F25	Best	2.80 × 10^3^	2.80 × 10^3^	2.90 × 10^3^	2.80 × 10^3^	2.83 × 10^3^	2.80 × 10^3^	2.90 × 10^3^	2.80 × 10^3^
	Ave	4.08 × 10^3^	4.87 × 10^3^	4.65 × 10^3^	4.90 × 10^3^	4.37 × 10^3^	4.15 × 10^3^	4.63 × 10^3^	4.43 × 10^3^
	Std	5.65 × 10^2^	6.02 × 10^2^	4.30 × 10^2^	1.36 × 10^3^	1.71 × 10^3^	8.57 × 10^2^	3.60 × 10^2^	1.32 × 10^3^
	Rank	1	7	6	8	3	2	5	4
F26	Best	3.19 × 10^3^	3.21 × 10^3^	3.20 × 10^3^	3.22 × 10^3^	3.23 × 10^3^	3.19 × 10^3^	3.20 × 10^3^	3.20 × 10^3^
	Ave	3.22 × 10^3^	3.24 × 10^3^	3.23 × 10^3^	3.26 × 10^3^	3.32 × 10^3^	3.23 × 10^3^	3.22 × 10^3^	3.25 × 10^3^
	Std	1.54 × 10^1^	2.11 × 10^1^	1.47 × 10^1^	2.61 × 10^1^	6.07 × 10^1^	1.35 × 10^1^	1.19 × 10^1^	2.52 × 10^1^
	Rank	2	5	4	7	8	3	1	6
F27	Best	3.10 × 10^3^	3.10 × 10^3^	3.21 × 10^3^	3.10 × 10^3^	3.22 × 10^3^	3.20 × 10^3^	3.21 × 10^3^	3.21 × 10^3^
	Ave	3.19 × 10^3^	3.22 × 10^3^	3.26 × 10^3^	3.18 × 10^3^	3.29 × 10^3^	3.23 × 10^3^	3.25 × 10^3^	3.25 × 10^3^
	Std	4.52 × 10^1^	2.55 × 10^1^	3.46 × 10^1^	4.78 × 10^1^	2.65 × 10^1^	1.94 × 10^1^	3.54 × 10^1^	2.41 × 10^1^
	Rank	2	3	7	1	8	4	5	6
F28	Best	3.34 × 10^3^	3.34 × 10^3^	3.49 × 10^3^	3.39 × 10^3^	4.00 × 10^3^	3.38 × 10^3^	3.46 × 10^3^	3.42 × 10^3^
	Ave	3.59 × 10^3^	3.77 × 10^3^	3.86 × 10^3^	3.78 × 10^3^	4.48 × 10^3^	3.69 × 10^3^	3.77 × 10^3^	3.82 × 10^3^
	Std	1.80 × 10^2^	1.80 × 10^2^	1.82 × 10^2^	2.11 × 10^2^	2.69 × 10^2^	1.92 × 10^2^	1.61 × 10^2^	1.60 × 10^2^
	Rank	1	4	7	5	8	2	3	6
F29	Best	5.56 × 10^3^	5.47 × 10^3^	2.21 × 10^4^	5.67 × 10^3^	2.10 × 10^5^	6.13 × 10^3^	5.28 × 10^3^	5.56 × 10^3^
	Ave	8.85 × 10^3^	9.51 × 10^3^	1.94 × 10^5^	9.18 × 10^3^	1.80 × 10^6^	9.81 × 10^3^	1.45 × 10^4^	1.01 × 10^4^
	Std	3.25 × 10^3^	3.17 × 10^3^	1.76 × 10^5^	2.72 × 10^3^	1.08 × 10^6^	2.99 × 10^3^	6.75 × 10^3^	4.25 × 10^3^
	Rank	1	3	7	2	8	4	6	5

**Table A3:** Comparison of statistical results derived from IFTTA and competitors for CEC 2017 (Dim = 50).

Function	Index	IFTTA	FTTA	RIME	MRFO	POEA	dFDBARO	DTSMA	RLTLBO
F1	Best	1.03 × 10^2^	3.21 × 10^3^	1.71 × 10^6^	1.00 × 10^2^	1.05 × 10^2^	1.14 × 10^6^	2.26 × 10^2^	3.45 × 10^6^
	Ave	7.41 × 10^3^	8.29 × 10^5^	4.29 × 10^6^	3.85 × 10^3^	2.75 × 10^3^	4.02 × 10^6^	2.91 × 10^6^	2.24 × 10^8^
	Std	8.54 × 10^3^	1.63 × 10^6^	1.48 × 10^6^	4.70 × 10^3^	2.75 × 10^3^	2.27 × 10^6^	8.83 × 10^6^	3.99 × 10^8^
	Rank	3	4	7	2	1	6	5	8
F2	Best	5.72 × 10^4^	5.55 × 10^4^	4.01 × 10^4^	6.12 × 10^4^	1.05 × 10^3^	7.00 × 10^4^	3.23 × 10^4^	3.80 × 10^4^
	Ave	9.73 × 10^4^	1.07 × 10^5^	8.56 × 10^4^	8.71 × 10^4^	2.91 × 10^3^	1.02 × 10^5^	7.50 × 10^4^	6.68 × 10^4^
	Std	2.10 × 10^4^	2.72 × 10^4^	2.13 × 10^4^	1.54 × 10^4^	1.01 × 10^3^	1.41 × 10^4^	2.01 × 10^4^	1.56 × 10^4^
	Rank	6	8	4	5	1	7	3	2
F3	Best	4.29 × 10^2^	4.71 × 10^2^	5.09 × 10^2^	4.09 × 10^2^	4.85 × 10^2^	4.96 × 10^2^	4.44 × 10^2^	5.12 × 10^2^
	Ave	5.10 × 10^2^	5.61 × 10^2^	6.17 × 10^2^	5.27 × 10^2^	6.23 × 10^2^	5.99 × 10^2^	5.90 × 10^2^	6.52 × 10^2^
	Std	5.59 × 10^1^	5.03 × 10^1^	5.00 × 10^1^	5.31 × 10^1^	5.39 × 10^1^	4.74 × 10^1^	4.40 × 10^1^	7.68 × 10^1^
	Rank	1	3	6	2	7	5	4	8
F4	Best	5.59 × 10^2^	6.42 × 10^2^	6.16 × 10^2^	6.87 × 10^2^	7.74 × 10^2^	6.53 × 10^2^	6.53 × 10^2^	7.18 × 10^2^
	Ave	6.60 × 10^2^	7.26 × 10^2^	6.79 × 10^2^	8.07 × 10^2^	8.35 × 10^2^	7.18 × 10^2^	7.22 × 10^2^	8.29 × 10^2^
	Std	6.02 × 10^1^	3.33 × 10^1^	3.28 × 10^1^	4.31 × 10^1^	3.21 × 10^1^	3.67 × 10^1^	4.47 × 10^1^	5.97 × 10^1^
	Rank	1	5	2	6	8	3	4	7
F5	Best	6.00 × 10^2^	6.01 × 10^2^	6.05 × 10^2^	6.08 × 10^2^	6.46 × 10^2^	6.03 × 10^2^	6.08 × 10^2^	6.15 × 10^2^
	Ave	6.00 × 10^2^	6.04 × 10^2^	6.13 × 10^2^	6.33 × 10^2^	6.62 × 10^2^	6.09 × 10^2^	6.19 × 10^2^	6.30 × 10^2^
	Std	1.90 × 10^−2^	2.17 × 10^0^	5.51 × 10^0^	1.19 × 10^1^	7.45 × 10^0^	4.19 × 10^0^	6.40 × 10^0^	8.09 × 10^0^
	Rank	1	2	4	7	8	3	5	6
F6	Best	8.11 × 10^2^	9.90 × 10^2^	9.13 × 10^2^	9.69 × 10^2^	1.11 × 10^3^	9.41 × 10^2^	9.42 × 10^2^	9.88 × 10^2^
	Ave	9.31 × 10^2^	1.14 × 10^3^	1.01 × 10^3^	1.29 × 10^3^	1.44 × 10^3^	1.07 × 10^3^	1.03 × 10^3^	1.20 × 10^3^
	Std	6.78 × 10^1^	1.20 × 10^2^	4.48 × 10^1^	1.70 × 10^2^	1.03 × 10^2^	7.19 × 10^1^	5.04 × 10^1^	1.31 × 10^2^
	Rank	1	5	2	7	8	4	3	6
F7	Best	8.57 × 10^2^	9.55 × 10^2^	9.04 × 10^2^	9.89 × 10^2^	1.09 × 10^3^	9.46 × 10^2^	9.42 × 10^2^	9.97 × 10^2^
	Ave	9.57 × 10^2^	1.01 × 10^3^	9.91 × 10^2^	1.12 × 10^3^	1.15 × 10^3^	1.02 × 10^3^	1.03 × 10^3^	1.11 × 10^3^
	Std	6.90 × 10^1^	3.45 × 10^1^	4.48 × 10^1^	6.07 × 10^1^	3.53 × 10^1^	3.85 × 10^1^	4.50 × 10^1^	5.31 × 10^1^
	Rank	1	3	2	7	8	4	5	6
F8	Best	9.19 × 10^2^	2.42 × 10^3^	1.69 × 10^3^	3.94 × 10^3^	6.66 × 10^3^	2.69 × 10^3^	2.98 × 10^3^	3.20 × 10^3^
	Ave	1.20 × 10^3^	6.12 × 10^3^	5.27 × 10^3^	1.03 × 10^4^	1.02 × 10^4^	6.26 × 10^3^	9.30 × 10^3^	1.17 × 10^4^
	Std	3.35 × 10^2^	2.20 × 10^3^	2.72 × 10^3^	2.73 × 10^3^	1.61 × 10^3^	1.76 × 10^3^	4.63 × 10^3^	5.17 × 10^3^
	Rank	1	3	2	7	6	4	5	8
F9	Best	4.54 × 10^3^	4.62 × 10^3^	5.99 × 10^3^	5.12 × 10^3^	7.04 × 10^3^	4.85 × 10^3^	5.96 × 10^3^	6.66 × 10^3^
	Ave	5.85 × 10^3^	7.04 × 10^3^	7.39 × 10^3^	7.41 × 10^3^	9.26 × 10^3^	6.23 × 10^3^	8.57 × 10^3^	1.15 × 10^4^
	Std	6.05 × 10^2^	9.12 × 10^2^	7.06 × 10^2^	8.47 × 10^2^	9.19 × 10^2^	6.87 × 10^2^	1.25 × 10^3^	2.11 × 10^3^
	Rank	1	3	4	5	7	2	6	8
F10	Best	1.15 × 10^3^	1.24 × 10^3^	1.36 × 10^3^	1.18 × 10^3^	1.27 × 10^3^	1.21 × 10^3^	1.29 × 10^3^	1.27 × 10^3^
	Ave	1.19 × 10^3^	1.32 × 10^3^	1.54 × 10^3^	1.24 × 10^3^	1.38 × 10^3^	1.28 × 10^3^	1.47 × 10^3^	1.37 × 10^3^
	Std	2.53 × 10^1^	4.31 × 10^1^	1.00 × 10^2^	2.80 × 10^1^	6.37 × 10^1^	4.04 × 10^1^	8.45 × 10^1^	5.93 × 10^1^
	Rank	1	4	8	2	6	3	7	5
F11	Best	3.74 × 10^5^	5.37 × 10^5^	1.88 × 10^7^	8.20 × 10^4^	3.38 × 10^6^	1.08 × 10^6^	1.60 × 10^6^	9.75 × 10^5^
	Ave	1.36 × 10^6^	4.58 × 10^6^	8.36 × 10^7^	1.38 × 10^6^	3.26 × 10^7^	5.09 × 10^6^	8.80 × 10^6^	6.24 × 10^6^
	Std	9.33 × 10^5^	5.14 × 10^6^	4.74 × 10^7^	9.12 × 10^5^	1.59 × 10^7^	2.73 × 10^6^	4.67 × 10^6^	4.39 × 10^6^
	Rank	1	3	8	2	7	4	6	5
F12	Best	1.40 × 10^3^	1.63 × 10^3^	3.95 × 10^4^	1.44 × 10^3^	2.16 × 10^4^	1.85 × 10^3^	2.48 × 10^3^	3.57 × 10^3^
	Ave	7.07 × 10^3^	8.51 × 10^3^	2.02 × 10^5^	6.61 × 10^3^	8.67 × 10^4^	5.57 × 10^3^	2.34 × 10^4^	9.95 × 10^3^
	Std	7.02 × 10^3^	7.54 × 10^3^	1.46 × 10^5^	6.54 × 10^3^	6.28 × 10^4^	4.04 × 10^3^	1.25 × 10^4^	5.89 × 10^3^
	Rank	3	4	8	2	7	1	6	5
F13	Best	5.54 × 10^3^	8.22 × 10^3^	3.99 × 10^4^	3.47 × 10^3^	5.34 × 10^3^	2.92 × 10^3^	4.42 × 10^4^	3.45 × 10^3^
	Ave	7.86 × 10^4^	1.36 × 10^5^	2.31 × 10^5^	5.94 × 10^4^	1.02 × 10^5^	6.05 × 10^4^	1.85 × 10^5^	5.02 × 10^4^
	Std	7.53 × 10^4^	1.48 × 10^5^	1.36 × 10^5^	4.40 × 10^4^	5.84 × 10^4^	4.16 × 10^4^	1.20 × 10^5^	4.79 × 10^4^
	Rank	4	6	8	2	5	3	7	1
F14	Best	1.57 × 10^3^	1.72 × 10^3^	1.52 × 10^4^	1.58 × 10^3^	1.09 × 10^4^	1.70 × 10^3^	1.93 × 10^3^	1.94 × 10^3^
	Ave	9.28 × 10^3^	9.48 × 10^3^	4.99 × 10^4^	8.59 × 10^3^	3.83 × 10^4^	6.91 × 10^3^	1.97 × 10^4^	1.22 × 10^4^
	Std	7.60 × 10^3^	6.79 × 10^3^	2.76 × 10^4^	6.50 × 10^3^	2.40 × 10^4^	4.92 × 10^3^	1.05 × 10^4^	6.44 × 10^3^
	Rank	3	4	8	2	7	1	6	5
F15	Best	2.22 × 10^3^	2.22 × 10^3^	2.12 × 10^3^	2.41 × 10^3^	2.61 × 10^3^	2.19 × 10^3^	2.36 × 10^3^	2.36 × 10^3^
	Ave	3.38 × 10^3^	3.47 × 10^3^	3.56 × 10^3^	3.35 × 10^3^	3.96 × 10^3^	3.16 × 10^3^	3.19 × 10^3^	3.05 × 10^3^
	Std	4.99 × 10^2^	4.77 × 10^2^	4.56 × 10^2^	4.34 × 10^2^	7.09 × 10^2^	4.18 × 10^2^	5.08 × 10^2^	3.56 × 10^2^
	Rank	5	6	7	4	8	2	3	1
F16	Best	2.00 × 10^3^	2.46 × 10^3^	2.62 × 10^3^	2.49 × 10^3^	2.57 × 10^3^	2.18 × 10^3^	2.37 × 10^3^	2.39 × 10^3^
	Ave	2.84 × 10^3^	3.20 × 10^3^	3.21 × 10^3^	3.07 × 10^3^	3.53 × 10^3^	2.82 × 10^3^	3.01 × 10^3^	2.94 × 10^3^
	Std	3.34 × 10^2^	3.62 × 10^2^	3.65 × 10^2^	3.10 × 10^2^	3.56 × 10^2^	2.70 × 10^2^	2.54 × 10^2^	3.04 × 10^2^
	Rank	2	6	7	5	8	1	4	3
F17	Best	1.11 × 10^5^	4.84 × 10^4^	4.18 × 10^5^	4.52 × 10^4^	1.49 × 10^5^	9.99 × 10^4^	2.79 × 10^5^	6.17 × 10^4^
	Ave	1.05 × 10^6^	5.95 × 10^5^	3.78 × 10^6^	5.55 × 10^5^	9.01 × 10^5^	7.74 × 10^5^	2.26 × 10^6^	6.12 × 10^5^
	Std	9.61 × 10^5^	7.18 × 10^5^	2.53 × 10^6^	4.85 × 10^5^	5.38 × 10^5^	6.74 × 10^5^	1.38 × 10^6^	3.79 × 10^5^
	Rank	6	2	8	1	5	4	7	3
F18	Best	1.92 × 10^3^	2.15 × 10^3^	3.24 × 10^3^	1.97 × 10^3^	5.32 × 10^4^	2.13 × 10^3^	2.15 × 10^3^	3.06 × 10^3^
	Ave	1.77 × 10^4^	1.82 × 10^4^	5.51 × 10^4^	1.58 × 10^4^	6.96 × 10^5^	1.49 × 10^4^	1.94 × 10^4^	2.04 × 10^4^
	Std	1.08 × 10^4^	1.23 × 10^4^	4.23 × 10^4^	9.42 × 10^3^	3.77 × 10^5^	8.67 × 10^3^	1.69 × 10^4^	9.05 × 10^3^
	Rank	3	4	7	2	8	1	5	6
F19	Best	2.20 × 10^3^	2.41 × 10^3^	2.36 × 10^3^	2.23 × 10^3^	2.43 × 10^3^	2.34 × 10^3^	2.44 × 10^3^	2.57 × 10^3^
	Ave	2.94 × 10^3^	3.11 × 10^3^	3.18 × 10^3^	3.15 × 10^3^	3.16 × 10^3^	2.93 × 10^3^	3.11 × 10^3^	3.04 × 10^3^
	Std	3.16 × 10^2^	3.36 × 10^2^	3.48 × 10^2^	3.18 × 10^2^	2.71 × 10^2^	2.42 × 10^2^	3.65 × 10^2^	2.73 × 10^2^
	Rank	2	4	8	6	7	1	5	3
F20	Best	2.35 × 10^3^	2.40 × 10^3^	2.40 × 10^3^	2.46 × 10^3^	2.58 × 10^3^	2.41 × 10^3^	2.42 × 10^3^	2.43 × 10^3^
	Ave	2.46 × 10^3^	2.52 × 10^3^	2.49 × 10^3^	2.55 × 10^3^	2.67 × 10^3^	2.48 × 10^3^	2.50 × 10^3^	2.51 × 10^3^
	Std	7.21 × 10^1^	4.59 × 10^1^	3.96 × 10^1^	4.54 × 10^1^	5.32 × 10^1^	3.21 × 10^1^	4.82 × 10^1^	2.96 × 10^1^
	Rank	1	6	3	7	8	2	4	5
F21	Best	6.63 × 10^3^	7.18 × 10^3^	7.06 × 10^3^	2.30 × 10^3^	2.33 × 10^3^	2.32 × 10^3^	7.41 × 10^3^	2.34 × 10^3^
	Ave	8.19 × 10^3^	8.61 × 10^3^	9.21 × 10^3^	9.21 × 10^3^	1.02 × 10^4^	7.48 × 10^3^	9.85 × 10^3^	4.01 × 10^3^
	Std	7.47 × 10^2^	8.57 × 10^2^	7.98 × 10^2^	1.75 × 10^3^	2.75 × 10^3^	2.23 × 10^3^	1.07 × 10^3^	3.31 × 10^3^
	Rank	3	4	5	6	8	2	7	1
F22	Best	2.77 × 10^3^	2.93 × 10^3^	2.87 × 10^3^	2.88 × 10^3^	3.09 × 10^3^	2.85 × 10^3^	2.86 × 10^3^	2.93 × 10^3^
	Ave	2.88 × 10^3^	3.01 × 10^3^	2.96 × 10^3^	3.05 × 10^3^	3.25 × 10^3^	2.94 × 10^3^	2.93 × 10^3^	3.05 × 10^3^
	Std	6.71 × 10^1^	5.05 × 10^1^	5.66 × 10^1^	8.46 × 10^1^	8.12 × 10^1^	5.13 × 10^1^	3.71 × 10^1^	7.44 × 10^1^
	Rank	1	5	4	7	8	3	2	6
F23	Best	2.97 × 10^3^	3.06 × 10^3^	3.02 × 10^3^	3.06 × 10^3^	3.20 × 10^3^	3.01 × 10^3^	3.01 × 10^3^	3.04 × 10^3^
	Ave	3.06 × 10^3^	3.16 × 10^3^	3.11 × 10^3^	3.24 × 10^3^	3.38 × 10^3^	3.13 × 10^3^	3.08 × 10^3^	3.20 × 10^3^
	Std	7.17 × 10^1^	5.89 × 10^1^	4.86 × 10^1^	9.58 × 10^1^	9.26 × 10^1^	4.67 × 10^1^	3.72 × 10^1^	7.84 × 10^1^
	Rank	1	5	3	7	8	4	2	6
F24	Best	2.96 × 10^3^	3.02 × 10^3^	3.02 × 10^3^	3.01 × 10^3^	3.10 × 10^3^	3.06 × 10^3^	2.98 × 10^3^	3.10 × 10^3^
	Ave	3.05 × 10^3^	3.09 × 10^3^	3.09 × 10^3^	3.08 × 10^3^	3.17 × 10^3^	3.13 × 10^3^	3.07 × 10^3^	3.20 × 10^3^
	Std	3.43 × 10^1^	2.97 × 10^1^	3.70 × 10^1^	2.57 × 10^1^	4.48 × 10^1^	4.41 × 10^1^	3.81 × 10^1^	5.82 × 10^1^
	Rank	1	4	5	3	7	6	2	8
F25	Best	4.62 × 10^3^	2.96 × 10^3^	5.26 × 10^3^	2.90 × 10^3^	2.99 × 10^3^	2.99 × 10^3^	5.11 × 10^3^	3.74 × 10^3^
	Ave	5.36 × 10^3^	7.30 × 10^3^	6.05 × 10^3^	6.70 × 10^3^	4.58 × 10^3^	6.23 × 10^3^	5.96 × 10^3^	8.70 × 10^3^
	Std	6.34 × 10^2^	1.57 × 10^3^	4.77 × 10^2^	3.53 × 10^3^	2.57 × 10^3^	1.93 × 10^3^	4.52 × 10^2^	2.54 × 10^3^
	Rank	2	7	4	6	1	5	3	8
F26	Best	3.25 × 10^3^	3.30 × 10^3^	3.32 × 10^3^	3.37 × 10^3^	3.50 × 10^3^	3.32 × 10^3^	3.26 × 10^3^	3.36 × 10^3^
	Ave	3.42 × 10^3^	3.50 × 10^3^	3.50 × 10^3^	3.59 × 10^3^	3.89 × 10^3^	3.49 × 10^3^	3.43 × 10^3^	3.64 × 10^3^
	Std	8.39 × 10^1^	1.09 × 10^2^	9.23 × 10^1^	1.39 × 10^2^	2.39 × 10^2^	9.00 × 10^1^	8.81 × 10^1^	1.33 × 10^2^
	Rank	1	4	5	6	8	3	2	7
F27	Best	3.27 × 10^3^	3.28 × 10^3^	3.29 × 10^3^	3.26 × 10^3^	3.38 × 10^3^	3.29 × 10^3^	3.32 × 10^3^	3.37 × 10^3^
	Ave	3.31 × 10^3^	3.34 × 10^3^	3.36 × 10^3^	3.32 × 10^3^	3.47 × 10^3^	3.41 × 10^3^	3.54 × 10^3^	3.54 × 10^3^
	Std	1.59 × 10^1^	3.20 × 10^1^	4.14 × 10^1^	3.00 × 10^1^	5.42 × 10^1^	5.33 × 10^1^	4.28 × 10^2^	7.92 × 10^1^
	Rank	1	3	4	2	6	5	8	7
F28	Best	3.38 × 10^3^	3.74 × 10^3^	3.85 × 10^3^	3.62 × 10^3^	5.00 × 10^3^	3.60 × 10^3^	3.72 × 10^3^	3.76 × 10^3^
	Ave	3.88 × 10^3^	4.33 × 10^3^	4.68 × 10^3^	4.34 × 10^3^	6.31 × 10^3^	4.09 × 10^3^	4.32 × 10^3^	4.70 × 10^3^
	Std	3.83 × 10^2^	2.87 × 10^2^	3.49 × 10^2^	3.34 × 10^2^	5.54 × 10^2^	2.93 × 10^2^	3.28 × 10^2^	4.11 × 10^2^
	Rank	1	4	6	5	8	2	3	7
F29	Best	6.04 × 10^5^	6.94 × 10^5^	1.09 × 10^7^	7.02 × 10^5^	3.46 × 10^7^	8.17 × 10^5^	7.60 × 10^5^	5.54 × 10^5^
	Ave	9.33 × 10^5^	9.75 × 10^5^	2.36 × 10^7^	1.07 × 10^6^	5.80 × 10^7^	1.46 × 10^6^	2.10 × 10^6^	9.79 × 10^5^
	Std	2.12 × 10^5^	2.14 × 10^5^	7.57 × 10^6^	2.82 × 10^5^	1.56 × 10^7^	3.11 × 10^5^	8.83 × 10^5^	2.87 × 10^5^
	Rank	1	2	7	4	8	5	6	3

**Table A4:** Comparison of statistical results derived from IFTTA and competitors for CEC 2017 (Dim = 100).

Function	Index	IFTTA	FTTA	RIME	MRFO	POEA	dFDBARO	DTSMA	RLTLBO
F1	Best	7.08 × 10^3^	1.72 × 10^7^	5.90 × 10^7^	1.19 × 10^5^	6.59 × 10^5^	5.62 × 10^8^	5.02 × 10^8^	4.01 × 10^9^
	Ave	4.49 × 10^4^	8.38 × 10^7^	9.86 × 10^7^	4.47 × 10^5^	2.84 × 10^6^	1.97 × 10^9^	4.24 × 10^9^	1.57 × 10^10^
	Std	4.81 × 10^4^	6.51 × 10^7^	2.03 × 10^7^	3.04 × 10^5^	3.26 × 10^6^	7.43 × 10^8^	2.17 × 10^9^	5.27 × 10^9^
	Rank	1	4	5	2	3	6	7	8
F2	Best	2.59 × 10^5^	2.92 × 10^5^	4.00 × 10^5^	2.41 × 10^5^	6.52 × 10^4^	2.51 × 10^5^	2.87 × 10^5^	2.39 × 10^5^
	Ave	3.76 × 10^5^	4.62 × 10^5^	5.11 × 10^5^	2.86 × 10^5^	8.82 × 10^4^	3.03 × 10^5^	4.73 × 10^5^	3.05 × 10^5^
	Std	5.44 × 10^4^	7.92 × 10^4^	6.16 × 10^4^	2.76 × 10^4^	8.83 × 10^3^	1.97 × 10^4^	8.58 × 10^4^	2.56 × 10^4^
	Rank	5	6	8	2	1	3	7	4
F3	Best	5.98 × 10^2^	6.94 × 10^2^	7.93 × 10^2^	6.78 × 10^2^	8.21 × 10^2^	1.04 × 10^3^	7.61 × 10^2^	1.42 × 10^3^
	Ave	6.98 × 10^2^	8.81 × 10^2^	9.28 × 10^2^	8.03 × 10^2^	9.65 × 10^2^	1.31 × 10^3^	9.60 × 10^2^	2.42 × 10^3^
	Std	5.14 × 10^1^	7.95 × 10^1^	8.17 × 10^1^	6.04 × 10^1^	7.92 × 10^1^	1.76 × 10^2^	1.08 × 10^2^	5.49 × 10^2^
	Rank	1	3	4	2	6	7	5	8
F4	Best	7.35 × 10^2^	9.79 × 10^2^	8.84 × 10^2^	1.07 × 10^3^	1.33 × 10^3^	9.35 × 10^2^	9.46 × 10^2^	1.21 × 10^3^
	Ave	9.84 × 10^2^	1.16 × 10^3^	1.04 × 10^3^	1.29 × 10^3^	1.48 × 10^3^	1.15 × 10^3^	1.17 × 10^3^	1.41 × 10^3^
	Std	1.31 × 10^2^	7.90 × 10^1^	7.85 × 10^1^	7.61 × 10^1^	6.63 × 10^1^	8.31 × 10^1^	9.10 × 10^1^	1.01 × 10^2^
	Rank	1	4	2	6	8	3	5	7
F5	Best	6.00 × 10^2^	6.08 × 10^2^	6.22 × 10^2^	6.35 × 10^2^	6.68 × 10^2^	6.25 × 10^2^	6.31 × 10^2^	6.27 × 10^2^
	Ave	6.00 × 10^2^	6.16 × 10^2^	6.35 × 10^2^	6.55 × 10^2^	6.79 × 10^2^	6.35 × 10^2^	6.49 × 10^2^	6.54 × 10^2^
	Std	4.55 × 10^−2^	4.18 × 10^0^	6.20 × 10^0^	6.40 × 10^0^	4.03 × 10^0^	5.91 × 10^0^	8.01 × 10^0^	8.41 × 10^0^
	Rank	1	2	3	7	8	4	5	6
F6	Best	1.11 × 10^3^	1.73 × 10^3^	1.43 × 10^3^	2.17 × 10^3^	2.59 × 10^3^	1.66 × 10^3^	1.66 × 10^3^	2.03 × 10^3^
	Ave	1.43 × 10^3^	2.11 × 10^3^	1.64 × 10^3^	2.68 × 10^3^	3.01 × 10^3^	2.14 × 10^3^	1.92 × 10^3^	2.43 × 10^3^
	Std	1.50 × 10^2^	2.02 × 10^2^	1.27 × 10^2^	3.01 × 10^2^	2.24 × 10^2^	1.98 × 10^2^	1.81 × 10^2^	2.26 × 10^2^
	Rank	1	4	2	7	8	5	3	6
F7	Best	9.88 × 10^2^	1.28 × 10^3^	1.16 × 10^3^	1.40 × 10^3^	1.65 × 10^3^	1.25 × 10^3^	1.23 × 10^3^	1.44 × 10^3^
	Ave	1.30 × 10^3^	1.48 × 10^3^	1.36 × 10^3^	1.67 × 10^3^	1.90 × 10^3^	1.44 × 10^3^	1.46 × 10^3^	1.75 × 10^3^
	Std	1.47 × 10^2^	9.35 × 10^1^	8.06 × 10^1^	1.26 × 10^2^	9.49 × 10^1^	8.36 × 10^1^	1.05 × 10^2^	1.60 × 10^2^
	Rank	1	5	2	6	8	3	4	7
F8	Best	5.00 × 10^3^	1.52 × 10^4^	9.90 × 10^3^	2.05 × 10^4^	1.68 × 10^4^	1.84 × 10^4^	1.98 × 10^4^	3.99 × 10^4^
	Ave	1.20 × 10^4^	2.33 × 10^4^	2.80 × 10^4^	2.29 × 10^4^	3.50 × 10^4^	2.33 × 10^4^	3.66 × 10^4^	5.86 × 10^4^
	Std	5.26 × 10^3^	4.12 × 10^3^	9.27 × 10^3^	1.22 × 10^3^	1.19 × 10^4^	3.02 × 10^3^	1.12 × 10^4^	1.09 × 10^4^
	Rank	1	3	5	2	6	4	7	8
F9	Best	1.04 × 10^4^	1.32 × 10^4^	1.27 × 10^4^	1.16 × 10^4^	1.93 × 10^4^	1.27 × 10^4^	1.50 × 10^4^	1.92 × 10^4^
	Ave	1.30 × 10^4^	1.60 × 10^4^	1.67 × 10^4^	1.48 × 10^4^	2.21 × 10^4^	1.51 × 10^4^	1.96 × 10^4^	2.30 × 10^4^
	Std	1.21 × 10^3^	1.53 × 10^3^	1.82 × 10^3^	1.41 × 10^3^	1.38 × 10^3^	1.32 × 10^3^	1.79 × 10^3^	1.78 × 10^3^
	Rank	1	4	5	2	7	3	6	8
F10	Best	2.39 × 10^3^	5.59 × 10^3^	5.34 × 10^3^	6.28 × 10^3^	2.71 × 10^3^	1.41 × 10^4^	3.84 × 10^3^	3.69 × 10^3^
	Ave	5.69 × 10^3^	1.91 × 10^4^	7.41 × 10^3^	1.86 × 10^4^	3.26 × 10^3^	2.72 × 10^4^	7.34 × 10^3^	6.35 × 10^3^
	Std	3.62 × 10^3^	1.03 × 10^4^	1.34 × 10^3^	7.74 × 10^3^	3.48 × 10^2^	7.66 × 10^3^	2.95 × 10^3^	2.57 × 10^3^
	Rank	2	7	5	6	1	8	4	3
F11	Best	2.35 × 10^6^	1.16 × 10^7^	1.79 × 10^8^	2.96 × 10^6^	1.36 × 10^8^	6.06 × 10^7^	2.03 × 10^7^	1.48 × 10^8^
	Ave	1.13 × 10^7^	4.73 × 10^7^	7.13 × 10^8^	1.23 × 10^7^	4.40 × 10^8^	1.73 × 10^8^	2.26 × 10^8^	8.77 × 10^8^
	Std	1.00 × 10^7^	2.23 × 10^7^	2.99 × 10^8^	6.75 × 10^6^	1.63 × 10^8^	7.33 × 10^7^	1.93 × 10^8^	1.45 × 10^9^
	Rank	1	3	7	2	6	4	5	8
F12	Best	1.65 × 10^3^	4.04 × 10^3^	1.29 × 10^5^	1.70 × 10^3^	2.26 × 10^4^	9.39 × 10^3^	1.21 × 10^4^	1.30 × 10^4^
	Ave	7.70 × 10^3^	4.76 × 10^4^	4.80 × 10^5^	7.42 × 10^3^	5.54 × 10^4^	1.89 × 10^4^	3.30 × 10^5^	4.25 × 10^5^
	Std	7.37 × 10^3^	9.48 × 10^4^	1.29 × 10^6^	5.78 × 10^3^	1.94 × 10^4^	5.64 × 10^3^	1.02 × 10^6^	1.58 × 10^6^
	Rank	2	4	8	1	5	3	6	7
F13	Best	1.45 × 10^5^	1.86 × 10^5^	8.96 × 10^5^	1.56 × 10^5^	1.73 × 10^5^	2.02 × 10^5^	8.18 × 10^5^	2.29 × 10^5^
	Ave	6.64 × 10^5^	1.34 × 10^6^	4.38 × 10^6^	6.67 × 10^5^	7.87 × 10^5^	1.07 × 10^6^	2.57 × 10^6^	9.32 × 10^5^
	Std	3.33 × 10^5^	8.81 × 10^5^	1.93 × 10^6^	3.02 × 10^5^	3.30 × 10^5^	5.62 × 10^5^	1.36 × 10^6^	4.36 × 10^5^
	Rank	1	6	8	2	3	5	7	4
F14	Best	1.63 × 10^3^	1.97 × 10^3^	4.25 × 10^4^	1.75 × 10^3^	2.00 × 10^4^	2.33 × 10^3^	2.74 × 10^3^	2.77 × 10^3^
	Ave	3.60 × 10^3^	5.64 × 10^3^	2.40 × 10^5^	4.90 × 10^3^	4.55 × 10^4^	4.31 × 10^3^	4.70 × 10^4^	7.23 × 10^3^
	Std	2.71 × 10^3^	3.85 × 10^3^	7.85 × 10^5^	3.59 × 10^3^	1.75 × 10^4^	1.73 × 10^3^	6.59 × 10^4^	3.74 × 10^3^
	Rank	1	4	8	3	6	2	7	5
F15	Best	4.13 × 10^3^	3.97 × 10^3^	4.51 × 10^3^	3.82 × 10^3^	6.54 × 10^3^	4.41 × 10^3^	3.62 × 10^3^	4.25 × 10^3^
	Ave	5.90 × 10^3^	5.84 × 10^3^	6.56 × 10^3^	5.65 × 10^3^	9.23 × 10^3^	5.58 × 10^3^	5.56 × 10^3^	6.22 × 10^3^
	Std	7.08 × 10^2^	6.79 × 10^2^	8.22 × 10^2^	7.30 × 10^2^	1.49 × 10^3^	5.71 × 10^2^	7.97 × 10^2^	9.91 × 10^2^
	Rank	5	4	7	3	8	2	1	6
F16	Best	3.50 × 10^3^	3.67 × 10^3^	4.47 × 10^3^	3.60 × 10^3^	4.58 × 10^3^	3.43 × 10^3^	4.36 × 10^3^	4.28 × 10^3^
	Ave	4.78 × 10^3^	5.21 × 10^3^	5.50 × 10^3^	4.67 × 10^3^	5.95 × 10^3^	4.36 × 10^3^	5.49 × 10^3^	5.30 × 10^3^
	Std	5.59 × 10^2^	5.10 × 10^2^	5.69 × 10^2^	6.00 × 10^2^	7.14 × 10^2^	4.72 × 10^2^	5.26 × 10^2^	5.52 × 10^2^
	Rank	3	4	7	2	8	1	6	5
F17	Best	4.74 × 10^5^	4.63 × 10^5^	2.37 × 10^6^	3.86 × 10^5^	3.86 × 10^5^	3.28 × 10^5^	1.28 × 10^6^	3.64 × 10^5^
	Ave	1.46 × 10^6^	1.65 × 10^6^	6.45 × 10^6^	1.30 × 10^6^	1.33 × 10^6^	1.77 × 10^6^	4.59 × 10^6^	1.14 × 10^6^
	Std	6.99 × 10^5^	8.55 × 10^5^	2.79 × 10^6^	6.02 × 10^5^	4.53 × 10^5^	9.32 × 10^5^	2.50 × 10^6^	6.16 × 10^5^
	Rank	4	5	8	2	3	6	7	1
F18	Best	1.97 × 10^3^	2.33 × 10^3^	9.88 × 10^5^	2.02 × 10^3^	3.70 × 10^5^	2.17 × 10^3^	2.68 × 10^3^	2.37 × 10^3^
	Ave	5.93 × 10^3^	9.09 × 10^3^	8.13 × 10^6^	5.76 × 10^3^	4.46 × 10^6^	5.00 × 10^3^	1.56 × 10^4^	2.07 × 10^4^
	Std	4.75 × 10^3^	8.26 × 10^3^	4.87 × 10^6^	4.49 × 10^3^	2.58 × 10^6^	3.56 × 10^3^	1.08 × 10^4^	5.03 × 10^4^
	Rank	3	4	8	2	7	1	5	6
F19	Best	3.33 × 10^3^	3.60 × 10^3^	4.46 × 10^3^	3.24 × 10^3^	4.32 × 10^3^	3.61 × 10^3^	4.14 × 10^3^	4.38 × 10^3^
	Ave	4.92 × 10^3^	5.15 × 10^3^	5.37 × 10^3^	5.04 × 10^3^	5.30 × 10^3^	4.78 × 10^3^	5.38 × 10^3^	6.25 × 10^3^
	Std	6.72 × 10^2^	6.66 × 10^2^	5.23 × 10^2^	6.67 × 10^2^	5.52 × 10^2^	5.58 × 10^2^	5.97 × 10^2^	7.86 × 10^2^
	Rank	2	4	6	3	5	1	7	8
F20	Best	2.52 × 10^3^	2.89 × 10^3^	2.76 × 10^3^	2.73 × 10^3^	3.28 × 10^3^	2.71 × 10^3^	2.78 × 10^3^	2.95 × 10^3^
	Ave	2.75 × 10^3^	3.02 × 10^3^	2.92 × 10^3^	3.00 × 10^3^	3.56 × 10^3^	2.88 × 10^3^	2.95 × 10^3^	3.13 × 10^3^
	Std	1.52 × 10^2^	8.06 × 10^1^	8.81 × 10^1^	1.21 × 10^2^	1.18 × 10^2^	6.64 × 10^1^	9.94 × 10^1^	1.01 × 10^2^
	Rank	1	6	3	5	8	2	4	7
F21	Best	1.29 × 10^4^	1.42 × 10^4^	1.58 × 10^4^	1.51 × 10^4^	2.19 × 10^4^	1.49 × 10^4^	1.79 × 10^4^	4.00 × 10^3^
	Ave	1.58 × 10^4^	1.89 × 10^4^	1.92 × 10^4^	1.91 × 10^4^	2.50 × 10^4^	1.82 × 10^4^	2.24 × 10^4^	1.90 × 10^4^
	Std	1.07 × 10^3^	1.70 × 10^3^	1.43 × 10^3^	1.77 × 10^3^	1.50 × 10^3^	1.60 × 10^3^	2.12 × 10^3^	7.74 × 10^3^
	Rank	1	3	6	5	8	2	7	4
F22	Best	2.97 × 10^3^	3.16 × 10^3^	3.28 × 10^3^	3.29 × 10^3^	3.96 × 10^3^	3.27 × 10^3^	3.25 × 10^3^	3.48 × 10^3^
	Ave	3.15 × 10^3^	3.34 × 10^3^	3.41 × 10^3^	3.58 × 10^3^	4.31 × 10^3^	3.41 × 10^3^	3.36 × 10^3^	3.78 × 10^3^
	Std	5.99 × 10^1^	6.85 × 10^1^	7.81 × 10^1^	1.13 × 10^2^	1.85 × 10^2^	7.66 × 10^1^	5.14 × 10^1^	1.44 × 10^2^
	Rank	1	2	4	6	8	5	3	7
F23	Best	3.50 × 10^3^	3.90 × 10^3^	3.76 × 10^3^	3.95 × 10^3^	4.75 × 10^3^	3.92 × 10^3^	3.67 × 10^3^	4.18 × 10^3^
	Ave	3.75 × 10^3^	4.07 × 10^3^	4.05 × 10^3^	4.42 × 10^3^	5.25 × 10^3^	4.18 × 10^3^	3.85 × 10^3^	4.65 × 10^3^
	Std	1.40 × 10^2^	9.87 × 10^1^	1.14 × 10^2^	2.01 × 10^2^	2.88 × 10^2^	1.10 × 10^2^	7.37 × 10^1^	1.95 × 10^2^
	Rank	1	4	3	6	8	5	2	7
F24	Best	3.19 × 10^3^	3.37 × 10^3^	3.49 × 10^3^	3.34 × 10^3^	3.50 × 10^3^	3.72 × 10^3^	3.60 × 10^3^	3.92 × 10^3^
	Ave	3.31 × 10^3^	3.53 × 10^3^	3.63 × 10^3^	3.45 × 10^3^	3.82 × 10^3^	3.95 × 10^3^	3.97 × 10^3^	4.73 × 10^3^
	Std	6.39 × 10^1^	8.80 × 10^1^	7.43 × 10^1^	5.77 × 10^1^	1.01 × 10^2^	1.27 × 10^2^	2.56 × 10^2^	4.56 × 10^2^
	Rank	1	3	4	2	5	6	7	8
F25	Best	8.50 × 10^3^	1.24 × 10^4^	1.09 × 10^4^	3.80 × 10^3^	5.85 × 10^3^	8.20 × 10^3^	1.03 × 10^4^	1.52 × 10^4^
	Ave	1.11 × 10^4^	1.58 × 10^4^	1.31 × 10^4^	1.90 × 10^4^	1.77 × 10^4^	1.72 × 10^4^	1.19 × 10^4^	2.61 × 10^4^
	Std	1.45 × 10^3^	2.27 × 10^3^	9.86 × 10^2^	6.93 × 10^3^	9.26 × 10^3^	2.64 × 10^3^	9.59 × 10^2^	3.55 × 10^3^
	Rank	1	4	3	7	6	5	2	8
F26	Best	3.43 × 10^3^	3.45 × 10^3^	3.62 × 10^3^	3.63 × 10^3^	3.82 × 10^3^	3.62 × 10^3^	3.42 × 10^3^	3.85 × 10^3^
	Ave	3.54 × 10^3^	3.65 × 10^3^	3.82 × 10^3^	3.90 × 10^3^	4.46 × 10^3^	3.86 × 10^3^	3.55 × 10^3^	4.33 × 10^3^
	Std	5.82 × 10^1^	1.00 × 10^2^	1.12 × 10^2^	1.66 × 10^2^	3.38 × 10^2^	1.29 × 10^2^	6.14 × 10^1^	3.05 × 10^2^
	Rank	1	3	4	6	8	5	2	7
F27	Best	3.37 × 10^3^	3.48 × 10^3^	3.56 × 10^3^	3.43 × 10^3^	3.71 × 10^3^	4.01 × 10^3^	3.73 × 10^3^	4.85 × 10^3^
	Ave	3.43 × 10^3^	3.61 × 10^3^	3.71 × 10^3^	3.57 × 10^3^	3.93 × 10^3^	4.47 × 10^3^	6.87 × 10^3^	6.16 × 10^3^
	Std	3.18 × 10^1^	4.91 × 10^1^	6.01 × 10^1^	4.91 × 10^1^	1.62 × 10^2^	2.91 × 10^2^	3.43 × 10^3^	7.75 × 10^2^
	Rank	1	3	4	2	5	6	8	7
F28	Best	5.10 × 10^3^	5.92 × 10^3^	7.24 × 10^3^	5.24 × 10^3^	9.23 × 10^3^	5.65 × 10^3^	5.95 × 10^3^	6.83 × 10^3^
	Ave	6.69 × 10^3^	7.03 × 10^3^	8.80 × 10^3^	6.72 × 10^3^	1.17 × 10^4^	6.84 × 10^3^	7.25 × 10^3^	8.42 × 10^3^
	Std	8.13 × 10^2^	5.29 × 10^2^	6.43 × 10^2^	5.24 × 10^2^	1.06 × 10^3^	5.06 × 10^2^	7.61 × 10^2^	7.63 × 10^2^
	Rank	1	4	7	2	8	3	5	6
F29	Best	6.40 × 10^3^	1.02 × 10^4^	1.90 × 10^7^	1.75 × 10^4^	2.95 × 10^7^	1.41 × 10^5^	3.19 × 10^4^	1.23 × 10^5^
	Ave	1.62 × 10^4^	6.15 × 10^4^	8.27 × 10^7^	7.59 × 10^4^	9.36 × 10^7^	6.55 × 10^5^	4.65 × 10^5^	1.24 × 10^6^
	Std	6.24 × 10^3^	5.51 × 10^4^	4.31 × 10^7^	6.74 × 10^4^	4.65 × 10^7^	3.74 × 10^5^	4.98 × 10^5^	2.08 × 10^6^
	Rank	1	2	7	3	8	5	4	6
